# Association of Early Pregnancy Perfluoroalkyl and Polyfluoroalkyl Substance Exposure With Birth Outcomes

**DOI:** 10.1001/jamanetworkopen.2023.14934

**Published:** 2023-05-31

**Authors:** Yu Zhang, Vicente Mustieles, Qi Sun, Brent Coull, Thomas McElrath, Sheryl L. Rifas-Shiman, Leah Martin, Yang Sun, Yi-Xin Wang, Emily Oken, Andres Cardenas, Carmen Messerlian

**Affiliations:** 1 Department of Environmental Health, Harvard T. H. Chan School of Public Health, Boston, Massachusetts; 2 University of Granada, Center for Biomedical Research, Instituto de Investigación Biosanitaria Ibs, Consortium for Biomedical Research in Epidemiology and Public Health Grenada, Spain; 3 Department of Nutrition, Harvard T. H. Chan School of Public Health, Boston, Massachusetts; 4 Channing Division of Network Medicine, Department of Medicine, Brigham and Women's Hospital, Boston, Massachusetts; 5 Harvard Medical School, Boston, Massachusetts; 6 Department of Epidemiology, Harvard T. H. Chan School of Public Health, Boston, Massachusetts; 7 Department of Biostatistics, Harvard T. H. Chan School of Public Health, Boston, Massachusetts; 8 Department of Obstetrics and Gynecology, Brigham and Women's Hospital, Boston, Massachusetts; 9 Department of Population Medicine, Harvard Medical School and Harvard Pilgrim Health Care Institute, Boston, Massachusetts; 10 Department of Epidemiology and Population Health, Stanford University, Stanford, California; 11 Department of Obstetrics and Gynecology, Vincent Center for Reproductive Biology, Massachusetts General Hospital Fertility Center, Boston

## Abstract

**Importance:**

Prenatal perfluoroalkyl and polyfluoroalkyl substances (PFAS) have been linked to adverse birth outcomes. Previous research showed that higher folate concentrations are associated with lower blood PFAS concentrations in adolescents and adults. Further studies are needed to explore whether prenatal folate status mitigates PFAS-related adverse birth outcomes.

**Objective:**

To examine whether prenatal folate status modifies the negative associations between pregnancy PFAS concentrations, birth weight, and gestational age previously observed in a US cohort.

**Design, Setting, and Participants:**

In a prospective design, a prebirth cohort of mothers or pregnant women was recruited between April 1999 and November 2002, in Project Viva, a study conducted in eastern Massachusetts. Statistical analyses were performed from May 24 and October 25, 2022.

**Exposure:**

Plasma concentrations of 6 PFAS compounds were measured in early pregnancy (median gestational week, 9.6). Folate status was assessed through a food frequency questionnaire and measured in plasma samples collected in early pregnancy.

**Main Outcomes and Measures:**

Birth weight and gestational age, abstracted from delivery records; birth weight *z* score, standardized by gestational age and infant sex; low birth weight, defined as birth weight less than 2500 g; and preterm birth, defined as birth at less than 37 completed gestational weeks.

**Results:**

The cohort included a total of 1400 mother-singleton pairs. The mean (SD) age of the mothers was 32.21 (4.89) years. Most of the mothers were White (73.2%) and had a college degree or higher (69.1%). Early pregnancy plasma perfluorooctanoic acid concentration was associated with lower birth weight and birth weight *z* score only among mothers whose dietary folate intake (birth weight: β, −89.13 g; 95% CI, −166.84 to −11.42 g; birth weight *z* score: −0.13; 95% CI, −0.26 to −0.003) or plasma folate concentration (birth weight: −87.03 g; 95% CI, −180.11 to 6.05 g; birth weight *z* score: −0.14; 95% CI, −0.30 to 0.02) were below the 25th percentile (dietary: 660 μg/d, plasma: 14 ng/mL). No associations were found among mothers in the higher folate level groups, although the tests for heterogeneity did not reject the null. Associations between plasma perfluorooctane sulfonic acid and perfluorononanoate (PFNA) concentrations and lower birth weight, and between PFNA and earlier gestational age were noted only among mothers whose prenatal dietary folate intake or plasma folate concentration was in the lowest quartile range. No associations were found among mothers in higher folate status quartile groups.

**Conclusions and Relevance:**

In this large, US prebirth cohort, early pregnancy exposure to select PFAS compounds was associated with adverse birth outcomes only among mothers below the 25th percentile of prenatal dietary or plasma folate levels.

## Key Points

**Question:**

To what extent does prenatal folate status modify associations between early pregnancy perfluoroalkyl and polyfluoroalkyl substance (PFAS) exposure and birth outcomes?

**Findings:**

In this cohort study of 1400 mother-singleton pairs in the US, higher early pregnancy plasma PFAS concentrations were associated with lower birth weight and gestational age and a higher risk of low birth weight. These findings were observed only among mothers in the lowest quartile of prenatal folate status measured by dietary intake or plasma biomarker.

**Meaning:**

The findings of this study suggest that PFAS exposure may be associated with adverse birth outcomes only among mothers with low folate status in early pregnancy.

## Teaser

This cohort study examines the outcomes associated with folate status and exposure to perfluoroalkyl and polyfluoroalkyl substances in women who are pregnant.

## Introduction

Perfluoroalkyl and polyfluoroalkyl substances (PFAS) are a class of synthetic chemicals with diverse commercial and industrial applications, such as nonstick cookware, cosmetics, fire-fighting foams, and food packaging.^1^ The PFAS are extremely persistent in the environment and have been detected in populations worldwide.^2,3,4,5,6^ Exposure to PFAS has been associated with adverse reproductive end points and other poor health outcomes.^7,8,9^

Prenatal PFAS exposure has been linked to lower birth weight, shorter gestational age, and preterm birth in epidemiological studies,^10,11,12,13,14,15^ including the Project Viva, a large, prospective prebirth cohort in the US.^11^ Despite adverse birth outcomes associated with prenatal PFAS exposure, we are not aware of any study that has assessed potential modifiable factors that may reduce harmful outcomes of PFAS exposure in vulnerable populations. Previous studies reported inverse associations between folate and PFAS concentrations in a representative US population,^16^ and protective effects of folate on PFAS–lower antibody associations in children.^17^ Folic acid supplementation has been universally recommended to prevent neural tube defects.^18^ Folate plays a critical role in DNA synthesis and methylation.^19^ Folate and PFAS may share common transporters in the human body,^20,21,22,23,24,25,26,27,28^ thus resulting in potential biological interactions between folate and PFAS. This study aimed to examine whether prenatal folate status modifies the association between PFAS exposure and singleton birth outcomes.

## Methods

### Population

Mother-singleton pairs were recruited at their first prenatal visit between April 1999 and November 2002, in Project Viva, a large prebirth cohort in the US. Data analysis was performed from May 24 to October 25, 2022. Details on the Project Viva study design and population are described elsewhere.^29^ The institutional review board of Harvard Pilgrim Health Care approved the study protocol. Participants provided written informed consent. This study followed the Strengthening the Reporting of Observational Studies in Epidemiology (STROBE) reporting guideline for cohort studies.

In brief, sociodemographic characteristics, reproductive history, early pregnancy diet, and nutrient supplementation were assessed by questionnaires and interviews. Participants provided a nonfasting blood sample at recruitment (median gestational week, 9.6; IQR, 8.7-10.9).

There were 1400 mother-singleton pairs with plasma PFAS measurement, dietary folate intake, and birth outcome data. Among these participants, a subpopulation of 898 mothers had plasma folate concentrations quantified. We compared the study population with the original Project Viva cohort. The participant inclusion diagram can be found in eFigure 1 in Supplement 1.

### PFAS Measurements

The nonfasting blood samples obtained in early pregnancy were centrifuged and stored in non–PFAS-containing cryovial tubes in liquid nitrogen freezers (≤−130 °C). Plasma samples were sent to the Division of Laboratory Sciences at the Centers for Disease Control and Prevention for quantification of 8 PFAS compounds, including perfluorooctanoic acid (PFOA), perfluorooctane sulfonic acid (PFOS), perfluorohexane sulfonic acid, perfluorononanoate (PFNA), 2-(N-ethyl-perfluorooctane sulfonamido) acetate (EtFOSAA), 2-(N-methyl-perfluorooctane sulfonamido) acetate (MeFOSAA), perfluorodecanoic acid (PFDeA), and perfluorooctane sulfonamide (PFOSA), using online solid-phase extraction coupled to high-performance liquid chromatography isotope dilution tandem mass spectrometry.^30,31^ Limits of detection were 0.2 ng/mL for PFOS and 0.1 ng/mL for all other PFAS compounds. Concentrations below the limit of detection were imputed with the limit of detection divided by the square root of 2.^32,33^ Detection rates for PFDeA (47.52%) and PFOSA (10.93%) were low and were excluded because imputing values may incur bias.^33^ All other PFAS were detected in more than 99% of the samples.

### Folate Measurements

A semiquantitative food frequency questionnaire was used to assess diet from the last menstrual period of the index pregnancy to the time of study recruitment.^34,35^ An interview was conducted at recruitment to assess supplement intake for the 3-month period before the date of the index pregnancy detection. Pregnancy dietary folate equivalent (DFE) intake (micrograms per day) included both natural food folate and folic acid from fortification and supplement intake and was estimated by multiplying the frequency of intake of standard portion sizes of each food item or supplement times its folate or folic acid content, according to the Harvard nutrient composition database.^35^ To account for the variations in folate intake by total energy consumption, we adjusted the DFE intake for total energy intake via the residual method.^36^

Baseline plasma samples for a subpopulation were sent to Boston Children’s Hospital’s Clinical and Epidemiological Research Laboratory for folate quantification (to convert from nanograms per milliliter to millimoles per liter, multiply by 2.266) by an electrochemiluminescence binding assay (Elecsys Folate III, Roche Diagnostic). The assay is approved by the US Food and Drug Administration and has high reliability for plasma folate measurement (day-to-day imprecision <3%).

### Birth Outcome Measurements

Date of birth and birth weight in grams were abstracted from medical records. Gestational age was calculated using the date of the last menstrual period and corrected by ultrasonography (16-20 weeks) if the 2 estimates differed by more than 10 days (9%).^11^ We computed birth weight *z* score standardized by gestational age and infant sex, using a US reference.^37^ Preterm birth was defined as birth less than 37 completed gestational weeks (259 days) and low birth weight as less than 2500 g.

### Covariates

Data on maternal characteristics were collected by questionnaires and interviews at recruitment. Demographic and socioeconomic characteristics included maternal age, maternal and paternal educational level, self-reported maternal race and ethnicity, and annual household income. Maternal prepregnancy body mass index was calculated by self-reported weight in kilograms divided by the square of self-reported height in meters. Smoking status was grouped as ever smokers, former smokers, and early pregnancy smokers. Reproductive history included parity and infertility history defined as having tried for more than 12 months (if age <35 years) or 6 months (if age ≥35 years) to achieve the index pregnancy or having a diagnosis of infertility or claims for infertility treatments and/or prescriptions in medical records.^38,39^ Index pregnancy was self-reported as planned or unplanned. Breastfeeding history was estimated using parity and breastfeeding of the index birth as previously described.^11^ We calculated the Alternative Healthy Eating Index for Pregnancy score as a measure of diet quality in early pregnancy.^40,41^

### Statistical Analysis

#### Primary Analyses

The study population was grouped by folate quartiles of DFE intake or plasma folate concentrations. We conducted descriptive analyses on population characteristics and PFAS distributions by folate groups. Spearman correlation coefficients of 6 PFAS concentrations, DFE intake, and plasma folate concentration were calculated.

Plasma PFAS concentrations were log-2 transformed to reduce the influence of outliers and facilitate comparison with previous studies.^12,14,42^ Multivariable linear regression models were used to assess differences in the continuous birth outcomes—gestational age (days), birth weight (grams), and birth weight *z* score, per doubling of plasma PFAS concentrations—by DFE intake or plasma folate concentration groups. Adjusted covariates were selected a priori using a directed acyclic graph and included maternal age, educational level, race and ethnicity, body mass index, smoking history, parity, breastfeeding history, infertility, planned pregnancy, infant sex, gestational age at recruitment, and Alternative Healthy Eating Index for Pregnancy score as well as paternal educational level and annual household income (eFigure 2 in Supplement 1).^11,43,44^

Quantile-based g computation (QGC) was used to assess the joint association of the total PFAS mixture with birth outcomes by folate groups.^45^ Quantile-based g computation estimated the differences in continuous birth outcomes per quartile increase in total PFAS mixture concentrations (lowest quartile as reference), adjusting for the above-mentioned covariates.

The associational estimates in each folate group were obtained from regression models with the interaction term of folate group × individual PFAS concentration. Likelihood ratio tests were used to test statistical significance of interaction terms in models with meaningful associations.

#### Supplemental Analyses

The 2 binary outcomes—preterm birth and low birth weight—were examined using logistic regressions (eMethods in Supplement 1). We additionally restricted DFE analyses to the subpopulation who had both DFE and plasma folate measurements to have comparable samples across both types of folate measures. We further adjusted for early pregnancy fish consumption since it is an important source of PFAS and associated with birth outcomes.^46^ Generalized additive models were used to explore nonlinear modification by folate (as DFE or plasma folate concentration) in individual PFAS and birth outcome associations (eMethods in Supplement 1).

All tests were 2-sided at an α = .05 significance threshold, except for the likelihood ratio tests of interaction terms (*P* < .20). All analyses were conducted in R, version 4.0.3 (R Foundation for Statistical Computing). In the linear and logistic regressions, we used multiple imputation with chained equations (MICE) to impute missing covariate values (missingness rates, 0.07%-6.93%) (eFigure 1 in Supplement 1) and obtained pooled regression coefficients with the MICE package (version 3.14.0). We used qgcompint (version 0.6.6) for quantile-based g computation models. Because of the difficulties in applying MICE in mixture models, missing values were imputed with the mean (continuous covariates) or the mode (categorical or binary covariates) in the mixture models and results by imputation methods were compared.

## Results

### Population

In the overall population of 1400 mothers or pregnant women, the mean (SD) age was 32.21 (4.89) years. Most mothers identified as White (1025 [73.2%]), had a college degree or higher (968 [69.1%]), had an annual household income greater than $70 000 (820 [58.6%]), planned for the pregnancy (879 [62.8%]), and reported periconception folic acid supplement use (957 [68.4%]) (Table 1). The incidence of preterm birth was 7.1% (n = 99) and the incidence of low birth weight was 4.6% (n = 64). Characteristics of the subpopulation were similar to those of the study population (Table 2). A comparison of the study population with the original Project Viva cohort can be found in eTable 1 and the eResults in Supplement 1.

**Table 1.** Characteristics of the Study Population in Project Viva

Characteristic	No. (%)					*P* value^a^
	Total population (N = 1400)	First quartile (n = 350)	Second quartile (n = 350)	Third quartile (n = 350)	Fourth quartile (n = 350)	
Dietary folate intake, range, μg/d	128.7-3497.2	128.7-660.4	660.4-936.2	936.2-1187.3	1187.3-3497.2	NA
Maternal age, mean (SD), y	32.21 (4.89)	30.67 (5.85)	32.20 (4.93)	32.82 (4.30)	33.15 (3.89)	<.001
Prepregnancy BMI, mean (SD)	24.78 (5.39)	25.99 (6.31)	24.66 (5.51)	24.31 (4.64)	24.16 (4.74)	<.001
AHEI-P score, mean (SD)^b^	60.46 (10.25)	55.67 (9.74)	58.53 (9.38)	63.09 (9.96)	64.56 (9.39)	<.001
Maternal race and ethnicity^c^						
Asian	68 (4.9)	22 (6.3)	16 (4.6)	16 (4.6)	14 (4.0)	<.001
Black	167 (11.9)	83 (23.7)	40 (11.4)	23 (6.6)	21 (6.0)	
Hispanic	87 (6.2)	38 (10.9)	17 (4.9)	22 (6.3)	10 (2.9)	
White	1025 (73.2)	183 (52.3)	265 (75.7)	285 (81.4)	292 (83.4)	
Other	53 (3.8)	24 (6.9)	12 (3.4)	4 (1.1)	13 (3.7)	
Maternal education ≥ college degree	968 (69.1)	173 (49.4)	246 (70.3)	261 (74.6)	288 (82.3)	<.001
Paternal education ≥ college degree^d^	888 (63.4)	144 (41.1)	213 (60.9)	257 (73.4)	274 (78.3)	<.001
Annual household income >$70 000^e^	820 (58.6)	149 (42.6)	201 (57.4)	222 (63.4)	248 (70.9)	<.001
Prenatal smoking^f^						
Never	932 (66.6)	228 (65.1)	225 (64.3)	233 (66.6)	246 (70.3)	<.001
Former	296 (21.1)	53 (15.1)	82 (23.4)	81 (23.1)	80 (22.9)	
Early pregnancy	168 (12.0)	68 (19.4)	43 (12.3)	35 (10.0)	22 (6.3)	
Nulliparous	696 (49.7)	133 (38.0)	165 (47.1)	208 (59.4)	190 (54.3)	<.001
Planned pregnancy^g^	879 (62.8)	124 (35.4)	215 (61.4)	255 (72.9)	285 (81.4)	<.001
Infertility	306 (21.9)	40 (11.4)	73 (20.9)	82 (23.4)	111 (31.7)	<.001
Periconception folic acid supplement use^h^	957 (68.4)	79 (22.6)	230 (65.7)	305 (87.1)	343 (98.0)	<.001
Ever breastfeeding^i^	516 (36.9)	147 (42.0)	127 (36.3)	112 (32.0)	130 (37.1)	<.001
Female infant	685 (48.9)	175 (50.0)	170 (48.6)	166 (47.4)	174 (49.7)	.90
Gestational age, mean (SD), wk	39.49 (1.86)	39.30 (2.14)	39.56 (1.76)	39.72 (1.50)	39.36 (1.95)	.01
Birth weight, mean (SD), g	3486.7 (572.8)	3427.6 (637.6)	3503.6 (571.3)	3522.4 (493.6)	3493.1 (577.6)	.14
Birth weight *z* score, mean (SD)^j^	0.21 (0.95)	0.14 (1.03)	0.22 (0.96)	0.20 (0.90)	0.27 (0.90)	.38
Preterm birth	99 (7.1)	33 (9.4)	22 (6.3)	15 (4.3)	29 (8.3)	.04
Low birth weight	64 (4.6)	21 (6.0)	16 (4.6)	9 (2.6)	18 (5.1)	.16

Abbreviations: AHEI-P, Alternative Healthy Eating Index in Pregnancy; BMI, body mass index (calculated as weight in kilograms divided by height in meters squared); NA, not applicable.

^a^ *P* values obtained from χ^2^ tests for categorical variables and *t* tests for continuous variables across folate groups.

^b^ Higher AHEI-P score indicates better diet quality.

^c^ Race and ethnicity was self-reported in the questionnaire. American Indian or Alaska Native and other were collapsed into a single category to balance sample size.

^d^ Missingness: 97 (6.93%) for paternal educational level.

^e^ Missingness: 96 (6.86%) for household income.

^f^ Missingness: 4 (0.29%) for prenatal smoking.

^g^ Missingness: 27 (1.93%) for planned pregnancy.

^h^ Periconception folic acid supplement use was defined by any use of prenatal vitamin or folic acid supplement during gestational weeks 0 to 4. Missingness: 1.

^i^ Missingness: 78 (5.57%) for ever breastfeeding.

^j^ Birth weight *z* score standardized by gestational age and sex, using a US national reference.

**Table 2.** Characteristics of the Subpopulation in Project Viva

Characteristic	No. (%)					*P* value^a^
	Subpopulation (n = 898)	First quartile (n = 225)	Second quartile (n = 225)	Third quartile (n = 224)	Fourth quartile (n = 224)	
Plasma folate concentration range, ng/mL	7.08-359.80	7.08-14.01	14.01-18.92	18.92-28.48	28.48-359.80	
Dietary folate intake range, μg/d	128.7-2874.3	128.7-676.1	676.1-941.9	941.9-1194.3	1194.3-2874.3	
Maternal age, mean (SD), y	32.68 (4.62)	32.25 (5.57)	32.91 (4.23)	32.71 (4.46)	32.86 (4.06)	.42
Prepregnancy BMI, mean (SD)	24.76 (5.25)	25.37 (6.13)	24.39 (5.21)	24.71 (5.14)	24.59 (4.35)	.22
AHEI-P score, mean (SD)^b^	61.10 (10.28)	59.91 (10.93)	60.72 (10.19)	61.96 (10.03)	61.81 (9.86)	.11
Maternal race and ethnicity^c^						
Asian	39 (4.3)	15 (6.7)	8 (3.6)	8 (3.6)	8 (3.6)	.05
Black	86 (9.6)	28 (12.4)	25 (11.1)	22 (9.8)	11 (4.9)	
Hispanic	57 (6.3)	18 (8.0)	11 (4.9)	14 (6.2)	14 (6.2)	
White	678 (75.5)	150 (66.7)	173 (76.9)	175 (78.1)	180 (80.4)	
Other	38 (4.2)	14 (6.2)	8 (3.6)	5 (2.2)	11 (4.9)	
Maternal education ≥ college degree	663 (73.8)	154 (68.4)	164 (72.9)	170 (75.9)	175 (78.1)	.11
Paternal education ≥ college degree^d^	596 (66.4)	131 (58.2)	151 (67.1)	155 (69.2)	159 (71.0)	.006
Annual household income >$70 000	580 (64.6)	138 (61.3)	145 (64.4)	156 (69.6)	141 (62.9)	.07
Prenatal smoking						
Never	611 (68.0)	151 (67.1)	152 (67.6)	147 (65.6)	161 (71.9)	.19
Former	194 (21.6)	45 (20.0)	50 (22.2)	60 (26.8)	39 (17.4)	
Early pregnancy	93 (10.4)	29 (12.9)	23 (10.2)	17 (7.6)	24 (10.7)	
Nulliparous	431 (48.0)	103 (45.8)	99 (44.0)	113 (50.4)	116 (51.8)	.29
Planned pregnancy^e^	580 (64.6)	142 (63.1)	142 (63.1)	140 (62.5)	156 (69.6)	.27
Infertility	210 (23.4)	51 (22.7)	55 (24.4)	50 (22.3)	54 (24.1)	.94
Periconception folic acid supplement use^f^	629 (70.0)	132 (58.7)	152 (67.6)	169 (75.4)	176 (78.6)	<.001
Ever breastfeeding^g^	381 (42.4)	103 (45.8)	100 (44.4)	92 (41.1)	86 (38.4)	.22
Female infant	437 (48.7)	113 (50.2)	108 (48.0)	115 (51.3)	101 (45.1)	.56
Gestational age, mean (SD), wk	39.55 (1.79)	39.40 (1.88)	39.49 (1.84)	39.50 (2.01)	39.81 (1.35)	.09
Birth weight, mean (SD), g	3519.5 (559.2)	3451.6 (585.6)	3499.5 (573.0)	3513.2 (592.6)	3613.9 (467.1)	.02
Birth weight *z* score, mean (SD)^h^	0.26 (0.95)	0.17 (0.99)	0.22 (0.93)	0.27 (0.95)	0.37 (0.90)	.14
Preterm birth	56 (6.2)	24 (10.7)	12 (5.3)	15 (6.7)	5 (2.2)	.003
Low birth weight	34 (3.8)	14 (6.2)	10 (4.4)	9 (4.0)	1 (0.4)	.01

Abbreviations: AHEI-P, Alternative Healthy Eating Index in Pregnancy; BMI, body mass index (calculated as weight in kilograms divided by height in meters squared).

SI conversion: To convert plasma folate to millimoles per liter, multiply by 2.266.

^a^ *P* values obtained from χ^2^ tests for categorical variables and *t* tests for continuous variables across folate groups.

^b^ Higher AHEI-P score means better diet quality.

^c^ Race and ethnicity were self-reported in the questionnaire. American Indian or Alaskan Native and other were collapsed into a single category to balance sample size.

^d^ Missingness: 53 (5.90%) for paternal education.

^e^ Missingness: 14 (1.56%) for planned pregnancy.

^f^ Periconception folic acid supplement use was defined by any use of prenatal vitamin or folic acid supplement during gestational weeks 0 to 4. Missingness: 1.

^g^ Missingness: 17 (1.89%) for ever breastfeeding.

^h^ Birth weight *z* score standardized by gestational age and sex, using a US national reference.

Across DFE groups, mothers in the lowest quartile were more racially and ethnically diverse, less educated, reported lower annual household income, were more likely to smoke during pregnancy, and were less likely to be nulliparous, plan for this pregnancy, have infertility, and use periconception folic acid supplements, compared with the other 3 DFE quartile groups (Table 1). However, population characteristics were similar across quartile groups by pregnancy plasma folate concentrations, except that the lowest plasma quartile group had a higher proportion of mothers who were Black and of nonusers of periconception folic acid supplement than the other 3 groups (Table 2).

### PFAS and Folate Distributions

The 6 PFAS compounds were detected in more than 99% of the samples. Distributions of plasma concentrations of the 6 PFAS compounds were similar in the study population and the subpopulation with plasma folate measurements and were similar across folate groups (DFE or plasma) (eTable 2 and eTable 3 in Supplement 1). The median pregnancy DFE intake was 936.2 (IQR, 660.4-1187.3) μg/d, and the median plasma folate concentration was 18.92 (IQR, 14.01-28.48) ng/mL.

Correlation coefficients for the 6 PFAS compounds ranged between 0.18 (PFNA and EtFOSAA) and 0.74 (PFOA and PFOS). Dietary folate intake had a low correlation (*r* = 0.24) with plasma folate (eFigure 3 in Supplement 1). Negative correlations with DFE were noted for MeFOSAA (*r* = −0.10) and EtFOSAA (*r* = −0.12), and no other correlations were found (eFigure 3 in Supplement 1).

### Associations of PFAS With Birth Outcomes Across Folate Groups

**Figure 1.**
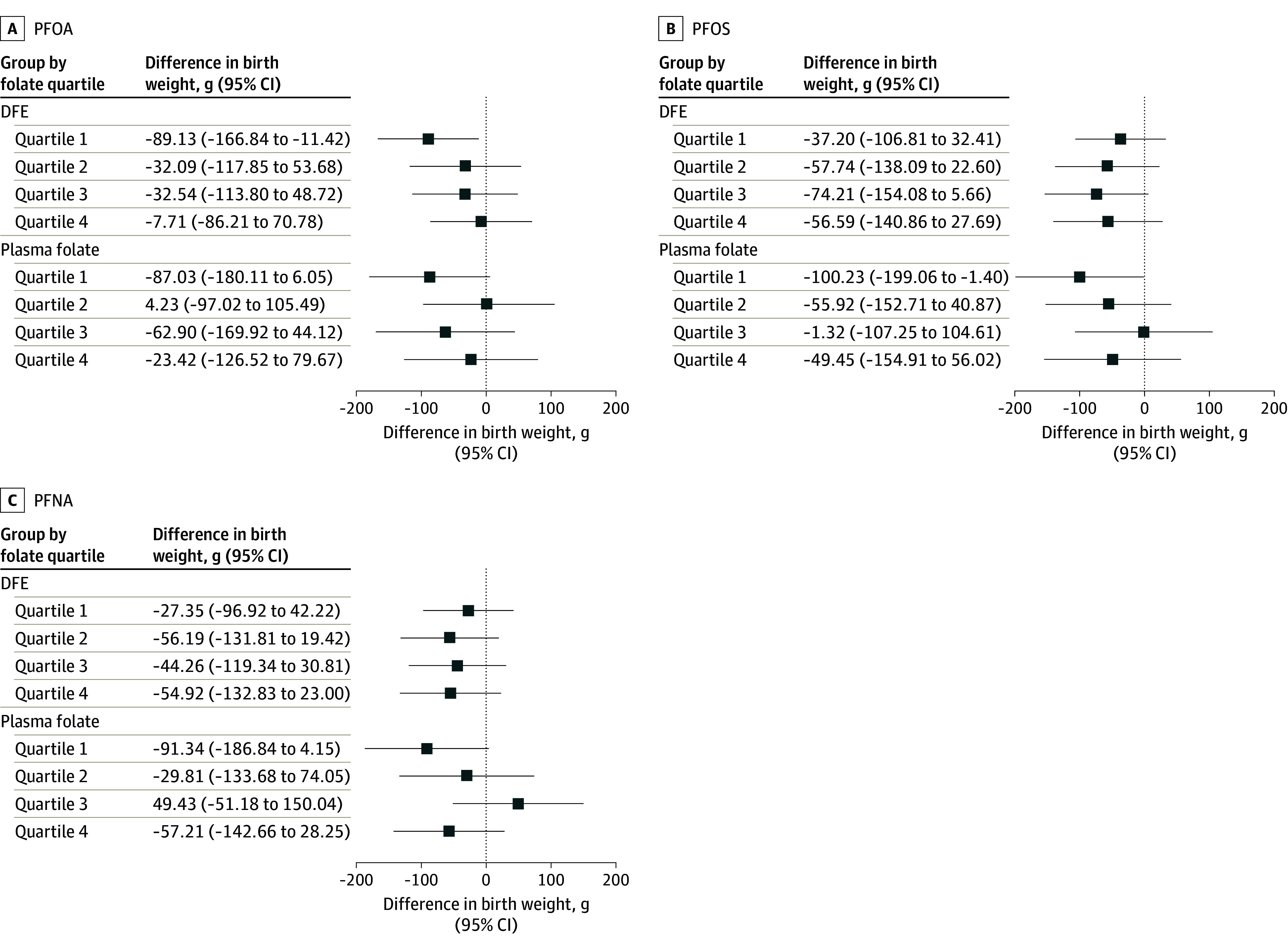
Associations of Early Pregnancy Plasma Polyfluoroalkyl and Polyfluoroalkyl Substances Concentrations With Birth Weight by Quartiles of Early Pregnancy Dietary Folate Intake (DFE) or Plasma Folate Concentrations Among Mother-Singleton Pairs in Project Viva Models were adjusted for maternal age (continuous), educational level (≥college graduate vs not college graduate), race and ethnicity (Asian, Black, Hispanic, White, other), prepregnancy body mass index (continuous), smoking history (never, former, early pregnancy), nulliparous (yes vs no), breastfeeding history (yes vs no), paternal educational level (≥college graduate vs not college graduate), annual household income (>$70 000 vs ≤$70 000), infertility (yes vs no), planned pregnancy (yes vs no), infant sex (male vs female), gestational age at recruitment (continuous), and Alternative Healthy Eating Index in Pregnancy score in early pregnancy (continuous). PFNA indicates perfluorononanoate; PFOA, perfluorooctanoic acid; and PFOS, perfluorooctane sulfonic acid.

Early pregnancy plasma PFOA, PFOS, and PFNA concentrations were associated with lower birth weight only among mothers in the lowest quartile of plasma folate concentrations (PFOA: −87.03 g; 95% CI, −180.11 to 6.05 g; PFOS: −100.23 g; 95% CI, −199.06 to −1.40 g; PFNA: −91.34 g; 95% CI, −186.84 to 4.15 g), and no associations were found for the other plasma folate quartile groups (PFOA: *P* = .54, PFOS: *P* = .60, PFNA: *P* = .20 for heterogeneity) (Figure 1). Higher plasma PFOA concentrations were associated with lower birth weight among mothers with the lowest DFE intake (β, −89.13 g; 95% CI, −166.84 to −11.42 g), but not among the other 3 DFE quartile groups (*P* = .49 for heterogeneity) (Figure 1). No associations with birth weight were found for the remaining PFAS compounds or PFAS mixture among either the DFE or plasma folate groups (eTable 4 in Supplement 1).

**Figure 2.**
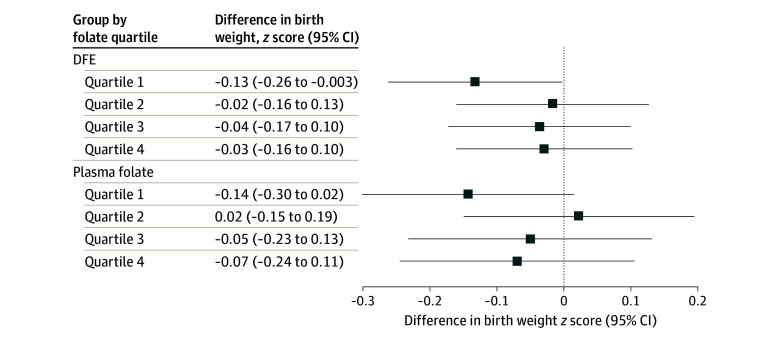
Associations of Early Pregnancy Plasma Perfluorooctanoic Acid Concentrations With Birth Weight *z* Score (Standardized by Gestational Age and Sex) by Quartiles of Early Pregnancy Dietary Folate Equivalent (DFE) Intake or Plasma Folate Concentrations Among Mother-Singleton Pairs in Project Viva Models were adjusted for maternal age (continuous), educational level (≥college graduate vs not college graduate), race and ethnicity (Asian, Black, Hispanic, White, other), prepregnancy body mass index (continuous), smoking history (never, former, early pregnancy), nulliparous (yes vs no), breastfeeding history (yes vs no), paternal educational level (≥college graduate vs not college graduate), annual household income (>$70 000 vs ≤$70 000), infertility (yes vs no), planned pregnancy (yes vs no), infant sex (male vs female), gestational age at recruitment (continuous), and Alternative Healthy Eating Index in Pregnancy score in early pregnancy (continuous).

Consistent with birth weight findings, plasma PFOA concentration was associated with a lower birth weight *z* score among the lowest DFE quartile (β, −0.13; 95% CI, −0.26 to −0.003) and the lowest plasma folate quartile groups (β, −0.14; 95% CI, −0.30 to 0.02), but not in the higher quartile groups (DFE: *P* = .57, plasma folate: *P* = .55 for heterogeneity) (Figure 2). The remaining PFAS compounds or PFAS mixture were not associated with birth weight *z* scores (eTable 5 in Supplement 1).

**Figure 3.**
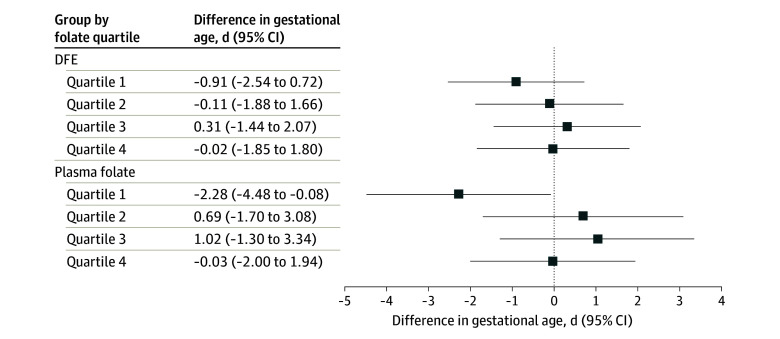
Associations of Early Pregnancy Plasma Perfluorononanoate Concentrations With Gestational Age by Quartiles of Early Pregnancy Dietary Folate Equivalent (DFE) Intake or Plasma Folate Concentrations Among Mother-Singleton Pairs in Project Viva Models were adjusted for maternal age (continuous), educational level (≥college graduate vs not college graduate), race and ethnicity (Asian, Black, Hispanic, White, other), prepregnancy body mass index (continuous), smoking history (never, former, early pregnancy), nulliparous (yes vs no), breastfeeding history (yes vs no), paternal educational level (≥college graduate vs not college graduate), annual household income (>$70 000 vs ≤$70 000), infertility (yes vs no), planned pregnancy (yes vs no), infant sex (male vs female), gestational age at recruitment (continuous), and Alternative Healthy Eating Index in Pregnancy score in early pregnancy (continuous).

Similarly, PFNA concentration was associated with lower gestational age among mothers in the lowest plasma folate quartile (β, −2.28 days; 95% CI, −4.48 to −0.08 days), while no associations were found for higher quartiles (*P* = .13 for heterogeneity) (Figure 3). No associations with gestational age were found for the remaining PFAS compounds or PFAS mixture in any of the other folate groups examined (eTable 6 in Supplement 1).

### Supplemental Analyses

Plasma PFOS concentration was associated with a higher risk of low birth weight among mothers in the lowest plasma folate quartile (odds ratio, 2.46; 95% CI, 1.05-5.80; *P* = .15 for heterogeneity) (eTable 7 in Supplement 1). There was no association between PFAS concentrations and preterm birth in any of the folate groups examined (eTable 7 and eTable 8 in Supplement 1).

Restricting the DFE analyses to the subpopulation in which we quantified plasma folate concentrations did not materially change the results from the total study population (eTable 9 in Supplement 1). Further adjusting for early pregnancy fish consumption only slightly attenuated the estimates (eTable 10 and eTable 11 in Supplement 1). The generalized additive models showed consistent modification by folate status with findings from primary analyses (eFigures 4-9 and eResults 2 in Supplement 1). Results obtained from single-value imputation were similar to those using MICE (eTables 12-16 in Supplement 1).

## Discussion

In this large, prospective prebirth cohort, we found that early pregnancy exposure to select PFAS compounds was associated with lower birth weight, birth weight *z* score, and gestational age, and higher risk of low birth weight only among mothers whose prenatal dietary folate intake or plasma folate concentrations were in the lowest quartile range (cutoff: <660 μg/d for dietary folate intake, <14 ng/mL for plasma folate), but not among mothers with higher dietary folate intake or plasma folate concentrations in early pregnancy. No consistent associations were found between PFAS exposure and risk of preterm birth or between PFAS mixture exposure and any of the birth outcomes in any of the folate groups.

Our findings of early pregnancy PFAS exposure and lower birth weight, lower gestational age, and higher risk of low birth weight among women with the lowest folate status are consistent with findings from several birth cohorts.^42,47,48,49,50,51,52^ A previous study with 1645 mothers from Project Viva reported inverse associations between early pregnancy plasma PFNA concentration and birth weight *z* score and gestational age in the total population, but the study did not examine birth weight as an outcome.^11^ In the present study, the associations between PFNA and lower gestational age were only seen in the lowest quartile group of plasma folate concentrations. A lower birth weight *z* score was associated with PFOA instead of PFNA only among mothers whose early pregnancy folate status was in the lowest quartile range. We additionally found associations of prenatal PFOA, PFOS, and PFNA concentrations with lower birth weight, and of PFOS with higher risk of low birth weight only in mothers with the lowest folate status. The early pregnancy plasma PFAS concentrations in our study population were similar to those in women of reproductive age in the general US population during similar time periods.^53^

To our knowledge, this is the first study to examine prenatal folate status as a modifiable factor for the associations between prenatal PFAS exposure and birth outcomes. We examined folate as both dietary intake and as a plasma biomarker, representing complementary folate assessments. The early pregnancy dietary folate intake crudely reflected folate intake from the last menstrual period up to the first trimester,^54^ which may not be representative of folate status over the entire pregnancy due to changes in food preferences in very early pregnancy. In contrast, plasma folate concentrations measure the internal dose of folate in circulation within a short time frame (days) before the time of sample collection in early pregnancy,^55^ when 86% of the Project Viva population reported using prenatal vitamin or folic acid supplements. Mothers in the lowest dietary folate quartile generally had lower socioeconomic status (SES) compared with mothers with higher folate status. However, SES in general was similar across quartile groups by plasma folate concentrations. Drawing similar conclusions from the 2 different folate measurements showing the PFAS associations with adverse birth outcomes were only found in mothers whose dietary folate intake or plasma folate levels were lower than the 25th percentiles supported our interpretation that these findings were not primarily due to residual confounding by SES. Furthermore, models were adjusted for parental educational level and household income. These results support our hypothesis that higher prenatal folate status could reduce or counteract PFAS exposure in birth outcomes. A recent study reported that higher PFAS concentrations were associated with lower antibody levels to rubella and mumps among adolescents with lower red blood cell folate levels but not in adolescents with higher folate levels in the US general population.^17^ In addition to neural tube defect prevention, these cumulative findings suggest that adequate folate status may counteract PFAS adverse effects and possibly the adverse effects associated with other environmental pollutants, as reported in experimental research.^56,57,58^

The mechanisms by which folate interacts with PFAS in birth outcomes are unclear and need further experimental investigation. Folate plays an important role in DNA synthesis and methylation, both critical to fetal growth.^59,60^ Sufficient folate supply in pregnancy has been associated with a decreased risk of low birth weight and small for gestational age.^61,62^ Evidence also shows that both folate and PFAS are substrates for several shared transporters in the human placenta, specifically the organic anion transporter family^22,63^; solute carrier family, such as folate receptor α; and the adenosine triphosphate–binding cassette family, including the breast cancer resistance protein and P-glycoprotein.^20,21,64,65,66^ Folate might compete with PFAS on shared transporters and thus reduce the placental transport of PFAS to the fetus. Furthermore, prenatal PFAS exposure is related to epigenetic changes in cord blood.^67^ Sufficient folate status might counteract PFAS-related epigenetic changes through its critical methyl-donor role in DNA methylation, thus mitigating the deleterious health effects.^68,69,70^

### Strengths and Limitations

This study examined prenatal folate as a modifiable factor for the associations between PFAS exposure and birth outcomes. The first strength of the study is its relatively large sample size, permitting stratified analyses by folate status. Second, PFAS concentrations were measured in plasma samples collected in early pregnancy, precluding potential confounding by hemodynamic changes occurring in late pregnancy.^11^ Third, dietary intake and plasma folate concentrations were both used to facilitate comparison and interpretation.

The study has limitations. Although both dietary and plasma folate status were evaluated, data on red blood cell folate, which reflects medium- to long-term folate intake, were not available.^55^ Mothers in Project Viva were folate replete; the 25th percentiles of the sample had dietary folate intake and plasma folate concentration above the recommended levels for neural tube defect prevention (dietary folate intake: 660 μg/d in Project Viva vs 600 μg/d in recommendations; plasma folate concentration: 14 ng/mL in Project Viva vs 11.26 ng/mL in recommendations).^18,71^ Further studies are needed to replicate the present findings in other populations with lower prenatal folate intake. Multiple comparisons were conducted that could result in inflated type I error. However, tests were based on prior hypotheses and consistent patterns of heterogeneity by folate status were identified that were unlikely due to chance. Dietary folate intake measured by food frequency questionnaires could have nondifferential measurement errors. However, food frequency questionnaires perform well in measuring the relative dietary intake (ie, ranks) in the population.^54^ While covariate adjustment included overall diet quality, residual confounding by diet cannot be ruled out. Because of the observational nature of this cohort study, causal effects of folate status on PFAS-related birth outcomes cannot be inferred. Selection bias is also possible given the differences in SES factors in the study sample with the original population in the Project Viva cohort. However, differences were subtle and several SES factors were included as covariates in the models. In addition, because of the complexity of the data analyses, we did not further examine sex-specific heterogeneities in the infants or nonlinear associations.

## Conclusions

In this prebirth cohort study, higher early pregnancy PFAS concentrations were associated with lower birth weight, birth weight *z* score, and gestational age, and higher risk of low birth weight only among mothers whose early pregnancy dietary folate intake or plasma folate levels were below the 25th percentiles. Findings suggest that mothers with the lowest folate status were more susceptible to PFAS-related adverse birth outcomes. Folate might be used as a PFAS prevention measure during preconception and pregnancy. If confirmed in other settings, the findings may have important implications for identifying vulnerable populations and implementing intervention studies.

## Notes

Supplement 1. **eTable 1.** Characteristics Across the Original Project Viva Cohort (N=2128), the Study Population (N=1400), and the Subpopulation (n=898) **eTable 2.** Distributions of Early Pregnancy Plasma Concentrations of Per- and Polyfluoroalkyl Substances in the Study Population and by Quartiles of Early Pregnancy Dietary Folate Intake Among Mother-Singleton Pairs in Project Viva **eTable 3.** Distributions of Early Pregnancy Plasma Concentrations of Per- and Polyfluoroalkyl Substances in the Study Population and by Quartiles of Early Pregnancy Plasma Folate Concentrations Among Mother-Singleton Pairs in Project Viva **eTable 4.** Differences (95% CI) in Birthweight (Grams) per Doubling of Early Pregnancy Plasma Concentrations of Per- and Polyfluoroalkyl Substances Across Quartile Groups by Early Pregnancy Dietary Folate Intake or Plasma Folate Concentrations Among Mother-Singleton Pairs in Project Viva **eTable 5.** Differences (95% CI) in Birthweight z Score per Doubling of Early Pregnancy Plasma Concentrations of Per- and Polyfluoroalkyl Substances Across Quartile Groups by Early Pregnancy Dietary Folate Intake or Plasma Folate Concentrations Among Mother-Singleton Pairs in Project Viva **eTable 6.** Differences (95% CI) in Gestational Age per Doubling of Early Pregnancy Plasma Concentrations of Per- and Polyfluoroalkyl Substances Across Quartile Groups by Early Pregnancy Dietary Folate Intake or Plasma Folate Concentrations Among Mother-Singleton Pairs in Project Viva **eTable 7.** Odds Ratios (95% CI) for Low Birthweight per Doubling of Early Pregnancy Plasma Concentrations of Per- and Polyfluoroalkyl Substances Across Quartile Groups by Early Pregnancy Dietary Folate Intake or Plasma Folate Concentrations Among Mother-Singleton Pairs in Project Viva **eTable 8.** Odds Ratios (95% CI) for Preterm Birth per Doubling of Early Pregnancy Plasma Concentrations of Per- and Polyfluoroalkyl Substances Across Quartile Groups by Early Pregnancy Dietary Folate Intake or Plasma Folate Concentrations Among Mother-Singleton Pairs in Project Viva **eTable 9.** Associations Between Early Pregnancy Plasma Concentrations of Per- and Polyfluoroalkyl Substances and Birth Outcomes Across Quartile Groups by Early Pregnancy Dietary Folate Intake in the Subpopulation **eTable 10.** Associations Between Early Pregnancy Plasma Concentrations of Per- and Polyfluoroalkyl Substances and Birth Outcomes Across Quartile Groups by Early Pregnancy Dietary Folate Intake, Further Adjusting for Average Fish Consumption in Early Pregnancy **eTable 11.** Associations Between Early Pregnancy Plasma Concentrations of Per- and Polyfluoroalkyl Substances and Birth Outcomes Across Quartile Groups by Early Pregnancy Plasma Folate Concentrations, Further Adjusting for Average Fish Consumption in Early Pregnancy **eTable 12.** Differences (95% CI) in Birthweight (Grams) per Doubling of Early Pregnancy Plasma Concentrations of Per- and Polyfluoroalkyl Substances Across Quartile Groups by Early Pregnancy Dietary Folate Intake or Plasma Folate Concentrations Among Mother-Singleton Pairs in Project Viva, Using Single-Value Imputation to Account for Missingness **eTable 13.** Differences (95% CI) in Birthweight z Score per Doubling of Early Pregnancy Plasma Concentrations of Per- and Polyfluoroalkyl Substances Across Quartile Groups by Early Pregnancy Dietary Folate Intake or Plasma Folate Concentrations Among Mother-Singleton Pairs in Project Viva, Using Single-Value Imputation to Account for Missingness **eTable 14.** Differences (95% CI) in Gestational Age (Days) per Doubling of Early Pregnancy Plasma Concentrations of Per- and Polyfluoroalkyl Substances Across Quartile Groups by Early Pregnancy Dietary Folate Intake or Plasma Folate Concentrations Among Mother-Singleton Pairs in Project Viva, Using Single-Value Imputation to Account for Missingness **eTable 15.** Odds Ratios (95% CI) for Low Birthweight per Doubling of Early Pregnancy Plasma Concentrations of Per- and Polyfluoroalkyl Substances Across Quartile Groups by Early Pregnancy Dietary Folate Intake or Plasma Folate Concentrations Among Mother-Singleton Pairs in Project Viva, Using Single-Value Imputation to Account for Missingness **eTable 16.** Odds Ratios (95% CI) for Preterm Birth per Doubling of Early Pregnancy Plasma Concentrations of Per- and Polyfluoroalkyl Substances Across Quartile Groups by Early Pregnancy Dietary Folate Intake or Plasma Folate Concentrations Among Mother-Singleton Pairs in Project Viva, Using Single-Value Imputation to Account for Missingness **eFigure 1.** Participants’ Inclusion Diagram of Mother-Singleton Pairs in Project Viva **eFigure 2.** Directed Acyclic Graph Under the Null Hypothesis of PFAS-Birth Outcome Associations **eFigure 3.** Spearman Correlation Coefficient Matrix for Early Pregnancy Dietary Folate Intake and Plasma Concentrations of Per- and Polyfluoroalkyl Substances and Folate Among Mother-Singleton Pairs in Project Viva **eFigure 4.** Changes in the Beta Coefficients for the Relationships Between Log-2 Transformed Plasma Concentrations of Per- and Polyfluoroalkyl Substances With Birthweight Across Early Pregnancy Dietary Folate Equivalent Intake **eFigure 5.** Changes in the Beta Coefficients for the Relationships Between Log-2 Transformed Plasma Concentrations of Per- and Polyfluoroalkyl Substances With Birthweight Across Early Pregnancy Plasma Folate Concentrations **eFigure 6.** Changes in the Beta Coefficients for the Relationships Between Log-2 Transformed Plasma Concentrations of Per- and Polyfluoroalkyl Substances With Birthweight z Score Across Early Pregnancy Dietary Folate Equivalent Intake **eFigure 7.** Changes in the Beta Coefficients for the Relationships Between Log-2 Transformed Plasma Concentrations of Per- and Polyfluoroalkyl Substances With Birthweight z Score Across Early Pregnancy Plasma Folate Concentrations **eFigure 8.** Changes in Log (Odds Ratio) for Low Birthweight in Relation to Log-2 Transformed Plasma Concentrations of Per- and Polyfluoroalkyl Substances Across Early Pregnancy Plasma Folate Concentrations **eFigure 9.** Changes in the Beta Coefficients for the Relationships Between Log-2 Transformed Plasma Concentrations of Per- and Polyfluoroalkyl Substances With Gestational Age Across Early Pregnancy Plasma Folate Concentrations **eMethods.** Details on Analyses and Models **eResults.** Details on Comparisons and Models

Supplement 2.
Data Sharing Statement
