# Error in Figure 1

**DOI:** 10.1001/jamanetworkopen.2024.28181

**Published:** 2024-07-23

In the Original Investigation titled “Association of Early Pregnancy Perfluoroalkyl and Polyfluoroalkyl Substance Exposure With Birth Outcomes,”^1^ published May 31, 2023, there was an error in Figure 1. The difference and 95% CI values in panel A (PFOA) should have been presented in panel B (PFOS), those in panel B should have been presented in panel C (PFNA), and those in panel C should have been presented in panel A. This article has been corrected.^1^

## References

[1] Zhang Y, Mustieles V, Sun Q, . Association of early pregnancy perfluoroalkyl and polyfluoroalkyl substance exposure with birth outcomes. JAMA Netw Open. 2023;6(5):e2314934. doi:10.1001/jamanetworkopen.2023.14934 37256622 PMC10233420

