# Supplementary material for: Association of Early Pregnancy Perfluoroalkyl and Polyfluoroalkyl Substance Exposure With Birth Outcomes
Source: JAMA Netw Open. 2023 May 31;6(5):e2314934. doi: 10.1001/jamanetworkopen.2023.14934 (PMC10233420; doi:10.1001/jamanetworkopen.2023.14934)
Supplement: Supplement 1. — eTable 1. Characteristics Across the Original Project Viva Cohort (N=2128), the Study Population (N=1400), and the Subpopulation (n=898) eTable 2. Distributions of Early Pregnancy Plasma Concentrations of Per- and Polyfluoroalkyl Substances in the Study Population and by Quartiles of Early Pregnancy Dietary Folate Intake Among Mother-Singleton Pairs in Project Viva eTable 3. Distributions of Early Pregnancy Plasma Concentrations of Per- and Polyfluoroalkyl Substances in the Study Population and by Quartiles of Early Pregnancy Plasma Folate Concentrations Among Mother-Singleton Pairs in Project Viva eTable 4. Differences (95% CI) in Birthweight (Grams) per Doubling of Early Pregnancy Plasma Concentrations of Per- and Polyfluoroalkyl Substances Across Quartile Groups by Early Pregnancy Dietary Folate Intake or Plasma Folate Concentrations Among Mother-Singleton Pairs in Project Viva eTable 5. Differences (95% CI) in Birthweight z Score per Doubling of Early Pregnancy Plasma Concentrations of Per- and Polyfluoroalkyl Substances Across Quartile Groups by Early Pregnancy Dietary Folate Intake or Plasma Folate Concentrations Among Mother-Singleton Pairs in Project Viva eTable 6. Differences (95% CI) in Gestational Age per Doubling of Early Pregnancy Plasma Concentrations of Per- and Polyfluoroalkyl Substances Across Quartile Groups by Early Pregnancy Dietary Folate Intake or Plasma Folate Concentrations Among Mother-Singleton Pairs in Project Viva eTable 7. Odds Ratios (95% CI) for Low Birthweight per Doubling of Early Pregnancy Plasma Concentrations of Per- and Polyfluoroalkyl Substances Across Quartile Groups by Early Pregnancy Dietary Folate Intake or Plasma Folate Concentrations Among Mother-Singleton Pairs in Project Viva eTable 8. Odds Ratios (95% CI) for Preterm Birth per Doubling of Early Pregnancy Plasma Concentrations of Per- and Polyfluoroalkyl Substances Across Quartile Groups by Early Pregnancy Dietary Folate Intake or Plasma Folate Concentrations Among Mothe [file jamanetwopen-e2314934-s001.pdf]

Supplementary Online Content

Zhang Y, Mustieles V, Sun Q, et al. Association of early pregnancy perfluoroalkyl and polyfluoroalkyl substance exposure with birth outcomes. *JAMA Netw Open*. 2023;6(5):e2314934. doi:10.1001/jamanetworkopen.2023.14934

eTable 1. Characteristics Across the Original Project Viva Cohort (N=2128), the Study Population (N=1400), and the Subpopulation (n=898)

eTable 2. Distributions of Early Pregnancy Plasma Concentrations of Per- and Polyfluoroalkyl Substances in the Study Population and by Quartiles of Early Pregnancy Dietary Folate Intake Among Mother-Singleton Pairs in Project Viva

eTable 3. Distributions of Early Pregnancy Plasma Concentrations of Per- and Polyfluoroalkyl Substances in the Study Population and by Quartiles of Early Pregnancy Plasma Folate Concentrations Among Mother-Singleton Pairs in Project Viva

eTable 4. Differences (95% CI) in Birthweight (Grams) per Doubling of Early Pregnancy Plasma Concentrations of Per- and Polyfluoroalkyl Substances Across Quartile Groups by Early Pregnancy Dietary Folate Intake or Plasma Folate Concentrations Among Mother-Singleton Pairs in Project Viva

eTable 5. Differences (95% CI) in Birthweight z Score per Doubling of Early Pregnancy Plasma Concentrations of Per- and Polyfluoroalkyl Substances Across Quartile Groups by Early Pregnancy Dietary Folate Intake or Plasma Folate Concentrations Among Mother-Singleton Pairs in Project Viva

eTable 6. Differences (95% CI) in Gestational Age per Doubling of Early Pregnancy Plasma Concentrations of Per- and Polyfluoroalkyl Substances Across Quartile Groups by Early Pregnancy Dietary Folate Intake or Plasma Folate Concentrations Among Mother-Singleton Pairs in Project Viva

eTable 7. Odds Ratios (95% CI) for Low Birthweight per Doubling of Early Pregnancy Plasma Concentrations of Per- and Polyfluoroalkyl Substances Across Quartile Groups by Early Pregnancy Dietary Folate Intake or Plasma Folate Concentrations Among Mother-Singleton Pairs in Project Viva

eTable 8. Odds Ratios (95% CI) for Preterm Birth per Doubling of Early Pregnancy Plasma Concentrations of Per- and Polyfluoroalkyl Substances Across Quartile Groups by Early Pregnancy Dietary Folate Intake or Plasma Folate Concentrations Among Mother-Singleton Pairs in Project Viva

eTable 9. Associations Between Early Pregnancy Plasma Concentrations of Per- and Polyfluoroalkyl Substances and Birth Outcomes Across Quartile Groups by Early Pregnancy Dietary Folate Intake in the Subpopulation

eTable 10. Associations Between Early Pregnancy Plasma Concentrations of Per- and Polyfluoroalkyl Substances and Birth Outcomes Across Quartile Groups by Early Pregnancy Dietary Folate Intake, Further Adjusting for Average Fish Consumption in Early Pregnancy

eTable 11. Associations Between Early Pregnancy Plasma Concentrations of Per- and Polyfluoroalkyl Substances and Birth Outcomes Across Quartile Groups by Early Pregnancy Plasma Folate Concentrations, Further Adjusting for Average Fish Consumption in Early Pregnancy

eTable 12. Differences (95% CI) in Birthweight (Grams) per Doubling of Early Pregnancy Plasma Concentrations of Per- and Polyfluoroalkyl Substances Across Quartile Groups by Early Pregnancy Dietary Folate Intake or Plasma Folate Concentrations Among Mother-Singleton Pairs in Project Viva, Using Single-Value Imputation to Account for Missingness

eTable 13. Differences (95% CI) in Birthweight z Score per Doubling of Early Pregnancy Plasma Concentrations of Per- and Polyfluoroalkyl Substances Across Quartile Groups by Early Pregnancy Dietary Folate Intake or Plasma Folate Concentrations Among Mother-Singleton Pairs in Project Viva, Using Single-Value Imputation to Account for Missingness

eTable 14. Differences (95% CI) in Gestational Age (Days) per Doubling of Early Pregnancy Plasma Concentrations of Per- and Polyfluoroalkyl Substances Across Quartile Groups by Early Pregnancy Dietary Folate Intake or Plasma Folate Concentrations Among Mother-Singleton Pairs in Project Viva, Using Single-Value Imputation to Account for Missingness

eTable 15. Odds Ratios (95% CI) for Low Birthweight per Doubling of Early Pregnancy Plasma Concentrations of Per- and Polyfluoroalkyl Substances Across Quartile Groups by Early Pregnancy Dietary Folate Intake or Plasma Folate Concentrations Among Mother-Singleton Pairs in Project Viva, Using Single-Value Imputation to Account for Missingness

eTable 16. Odds Ratios (95% CI) for Preterm Birth per Doubling of Early Pregnancy Plasma Concentrations of Per- and Polyfluoroalkyl Substances Across Quartile Groups by Early Pregnancy Dietary Folate Intake or Plasma Folate Concentrations Among Mother-Singleton Pairs in Project Viva, Using Single-Value Imputation to Account for Missingness

eFigure 1. Participants' Inclusion Diagram of Mother-Singleton Pairs in Project Viva

eFigure 2. Directed Acyclic Graph Under the Null Hypothesis of PFAS-Birth Outcome Associations

eFigure 3. Spearman Correlation Coefficient Matrix for Early Pregnancy Dietary Folate Intake and Plasma Concentrations of Per- and Polyfluoroalkyl Substances and Folate Among Mother-Singleton Pairs in Project Viva

eFigure 4. Changes in the Beta Coefficients for the Relationships Between Log-2 Transformed Plasma Concentrations of Per- and Polyfluoroalkyl Substances With Birthweight Across Early Pregnancy Dietary Folate Equivalent Intake

eFigure 5. Changes in the Beta Coefficients for the Relationships Between Log-2 Transformed Plasma Concentrations of Per- and Polyfluoroalkyl Substances With Birthweight Across Early Pregnancy Plasma Folate Concentrations

eFigure 6. Changes in the Beta Coefficients for the Relationships Between Log-2 Transformed Plasma Concentrations of Per- and Polyfluoroalkyl Substances With Birthweight z Score Across Early Pregnancy Dietary Folate Equivalent Intake

eFigure 7. Changes in the Beta Coefficients for the Relationships Between Log-2 Transformed Plasma Concentrations of Per- and Polyfluoroalkyl Substances With Birthweight z Score Across Early Pregnancy Plasma Folate Concentrations

eFigure 8. Changes in Log (Odds Ratio) for Low Birthweight in Relation to Log-2 Transformed Plasma Concentrations of Per- and Polyfluoroalkyl Substances Across Early Pregnancy Plasma Folate Concentrations

eFigure 9. Changes in the Beta Coefficients for the Relationships Between Log-2 Transformed Plasma Concentrations of Per- and Polyfluoroalkyl Substances With Gestational Age Across Early Pregnancy Plasma Folate Concentrations

eMethods. Details on Analyses and Models

eResults. Details on Comparisons and Models

This supplementary material has been provided by the authors to give readers additional information about their work.

© 2023 Zhang Y et al. *JAMA Network Open.*

eTable 1. Characteristics across the original Project Viva cohort (N=2128), the study population (N=1400), and the subpopulation (N=898).

	Original Population of Project Viva N=2128	Study Population N=1400	Subpopulation N=898
Maternal age, years, mean (SD)	31.81 (5.22)	32.21 (4.89)	32.68 (4.62)
Pre-pregnancy BMI, kg/m^2, mean (SD)	24.89 (5.52)	24.78 (5.39)	24.76 (5.25)
AHEI-P score, mean (SD)	60.57 (10.24)	60.46 (10.25)	61.10 (10.28)
Maternal race and ethnicity			
White	1399 (65.7)	1025 (73.2)	678 (75.5)
Black	348 (16.4)	167 (11.9)	86 (9.6)
Hispanic	154 (7.2)	87 (6.2)	57 (6.3)
Asian	120 (5.6)	68 (4.9)	39 (4.3)
Other	83 (3.9)	53 (3.8)	38 (4.2)
NA	24 (1.1)	0	0
Maternal education ≥ college degree	1360 (63.9)	968 (69.1)	663 (73.8)
NA	24 (1.1)	0	0
Paternal education ≥ college degree	1229 (57.8)	888 (63.4)	596 (66.4)
NA	224 (10.5)	97 (6.9)	0
Annual household income > $70,000	1146 (53.9)	820 (58.6)	580 (64.6)
NA	254 (11.9)	96 (6.9)	0
Prenatal smoking			
Never smoker	1443 (67.8)	932 (66.6)	611 (68.0)
Former smoker	398 (18.7)	296 (21.1)	194 (21.6)
Early pregnancy smoker	266 (12.5)	168 (12.0)	93 (10.4)
NA	21 (1.0)	4 (0.3)	0
Nulliparous	1017 (47.8)	696 (49.7)	431 (48.0)
Planned pregnancy	1211 (56.9)	879 (62.8)	580 (64.6)
NA	114 (5.4)	27 (1.9)	14 (1.6)
Infertility	442 (20.8)	306 (21.9)	210 (23.4)
Periconception folic acid supplement user	1337 (62.8)	957 (68.4)	629 (70.0)
NA	50 (2.3)	1 (0.1)	1 (0.1)
Ever breastfeeding	766 (36.0)	516 (36.9)	381 (42.4)
NA	185 (8.7)	78 (5.6)	17 (1.9)
Female infant	1032 (48.5)	685 (48.9)	437 (48.7)
Gestational age, weeks, mean (SD)	39.42 (1.97)	39.49 (1.86)	39.55 (1.79)
Birthweight, grams, mean (SD)	3460.91 (592.39)	3486.7 (572.8)	3519.47 (559.17)
Birthweight z score, mean (SD)	0.17 (0.97)	0.21 (0.95)	0.26 (0.95)
Preterm birth	154 (7.2)	99 (7.1)	56 (6.2)
Low birthweight	111 (5.2)	64 (4.6)	34 (3.8)

Abbrev. SD, standard deviation; AHEI-P, alternative healthy eating index in pregnancy; BMI, body mass index; NA, missing value.

© 2023 Zhang Y et al. *JAMA Network Open.*

eTable 2. Distributions of early pregnancy plasma concentrations of per- and polyfluoroalkyl substances in the study population and by quartiles of early pregnancy dietary folate intake among mother-singleton pairs in Project Viva.

	Total Population N=1400		1st Quartile N=350	2nd Quartile N=350	3rd Quartile N=350	4th Quartile N=350
Chemical	Detection Rate (%)	Median (IQR), ng/mL	Median (IQR), ng/mL	Median (IQR), ng/mL	Median (IQR), ng/mL	Median (IQR), ng/mL
PFOA	100	5.8 (4.1, 7.9)	5.6 (3.92, 7.9)	5.9 (4.4, 7.7)	6 (4.6, 8)	5.5 (3.82, 8)
PFOS	99.9	25.2 (18.6, 34.4)	25.8 (19.3, 35)	26.4 (19.1, 35.6)	25.9 (18.4, 34.4)	23.8 (17.6, 32.6)
PFHxS	99.1	2.4 (1.6, 3.7)	2.25 (1.6, 3.48)	2.4 (1.63, 3.58)	2.5 (1.8, 3.9)	2.6 (1.6, 3.88)
PFNA	99.6	0.7 (0.5, 0.9)	0.6 (0.5, 0.9)	0.7 (0.5, 0.9)	0.7 (0.5, 0.9)	0.7 (0.5, 0.9)
MeFOSAA	100	1.9 (1.2, 3)	2.1 (1.3, 3.1)	2.1 (1.4, 3.6)	1.65 (1.2, 2.77)	1.8 (1.1, 2.7)
EtFOSAA	99.6	1.2 (0.7, 1.9)	1.3 (0.8, 2)	1.3 (0.8, 2.1)	1.1 (0.7, 1.7)	1 (0.6, 1.8)

Abbrev. IQR, interquartile range; PFOA, perfluorooctanoic acid; PFOS, perfluorooctane sulfonic acid; PFHxS, perfluorohexane sulfonic acid; PFNA, perfluorononanoate; MeFOSAA, 2-(N-methyl-perfluorooctane sulfonamido) acetate; EtFOSAA, 2-(N-ethyl-perfluorooctane sulfonamido) acetate.

© 2023 Zhang Y et al. *JAMA Network Open.*

eTable 3. Distributions of early pregnancy plasma concentrations of per- and polyfluoroalkyl substances in the subpopulation and by quartiles of early pregnancy plasma folate concentrations among mother-singleton pairs in Project Viva.

	Total Population N=898		1st Quartile N=225	2nd Quartile N=225	3rd Quartile N=224	4th Quartile N=224
Chemical	Detection Rate (%)	Median (IQR), ng/mL	Median (IQR), ng/mL	Median (IQR), ng/mL	Median (IQR), ng/mL	Median (IQR), ng/mL
PFOA	100	5.6 (4.03, 7.7)	5.7 (3.7, 8)	5.2 (3.9, 7.2)	5.6 (4.1, 7.6)	5.9 (4.4, 7.7)
PFOS	100	24.8 (18.3, 34.1)	25.3 (18.2, 35.4)	24 (18.1, 32.6)	25.5 (19.2, 34)	24.8 (18.2, 33)
PFHxS	99.2	2.4 (1.6, 3.7)	2.4 (1.5, 4.1)	2.2 (1.6, 3.7)	2.6 (1.8, 3.73)	2.6 (1.7, 3.6)
PFNA	99.1	0.6 (0.5, 0.9)	0.7 (0.5, 0.9)	0.6 (0.5, 0.9)	0.6 (0.5, 0.9)	0.7 (0.5, 0.9)
MeFOSAA	100	1.8 (1.2, 2.9)	1.9 (1.3, 2.8)	1.8 (1.2, 2.7)	1.8 (1.2, 3)	2 (1.3, 3.2)
EtFOSAA	99.7	1.1 (0.7, 1.9)	1.2 (0.7, 1.8)	1.1 (0.7, 1.8)	1.1 (0.775, 1.83)	1.1 (0.7, 1.92)

Abbrev. IQR, interquartile range; PFOA, perfluorooctanoic acid; PFOS, perfluorooctane sulfonic acid; PFHxS, perfluorohexane sulfonic acid; PFNA, perfluorononanoate; MeFOSAA, 2-(N-methyl-perfluorooctane sulfonamido) acetate; EtFOSAA, 2-(N-ethyl-perfluorooctane sulfonamido) acetate.

© 2023 Zhang Y et al. *JAMA Network Open.*

eTable 4. Differences (95% CI) in birthweight (grams) per doubling of early pregnancy plasma concentrations of per- and polyfluoroalkyl substances across quartile groups by early pregnancy dietary folate intake or plasma folate concentrations among mother-singleton pairs in Project Viva.

	Early Pregnancy Dietary Folate Intake			
	1st Quartile	2nd Quartile	3rd Quartile	4th Quartile
Chemical	Beta (95%CI) [a]	Beta (95%CI) [a]	Beta (95%CI) [a]	Beta (95%CI) [a]
PFOA	**-89.13 (-166.84, -11.42)**	-32.09 (-117.85, 53.68)	-32.54 (-113.80, 48.72)	-7.71 (-86.21, 70.78)
PFOS	-37.20 (-106.81, 32.41)	-57.74 (-138.09, 22.60)	-74.21 (-154.08, 5.66)	-56.59 (-140.86, 27.69)
PFHxS	-0.88 (-53.94, 52.17)	-31.74 (-89.79, 26.31)	1.54 (-46.55, 49.64)	4.42 (-49.53, 58.38)
PFNA	-27.35 (-96.92, 42.22)	-56.19 (-131.81, 19.42)	-44.26 (-119.34, 30.81)	-54.92 (-132.83, 23.00)
MeFOSAA	11.74 (-49.14, 72.62)	-15.63 (-72.74, 41.48)	-1.31 (-65.96, 63.33)	-53.46 (-113.24, 6.33)
EtFOSAA	28.88 (-24.72, 82.47)	-16.29 (-66.81, 34.23)	-24.53 (-76.62, 27.56)	-23.43 (-78.96, 32.10)
PFAS mixture [b]	22.10 (-57.99, 102.19)	-54.85 (-131.19, 21.49)	-30.45 (-109.85, 48.95)	-40.59 (-118.76, 37.58)
	Early Pregnancy Plasma Folate Concentrations			
	1st Quartile	2nd Quartile	3rd Quartile	4th Quartile
Chemical	Beta (95%CI) [a]	Beta (95%CI) [a]	Beta (95%CI) [a]	Beta (95%CI) [a]
PFOA	**-87.03 (-180.11, 6.05)**	4.23 (-97.02, 105.49)	-62.90 (-169.92, 44.12)	-23.42 (-126.52, 79.67)
PFOS	**-100.23 (-199.06, -1.40)**	-55.92 (-152.71, 40.87)	-1.32 (-107.25, 104.61)	-49.45 (-154.91, 56.02)
PFHxS	8.73 (-58.11, 75.56)	-44.03 (-104.66, 16.60)	24.73 (-38.46, 87.91)	-28.28 (-96.89, 40.33)
PFNA	**-91.34 (-186.84, 4.15)**	-29.81 (-133.68, 74.05)	49.43 (-51.18, 150.04)	-57.21 (-142.66, 28.25)
MeFOSAA	-60.14 (-140.90, 20.62)	-7.87 (-85.85, 70.11)	-32.32 (-109.17, 44.53)	6.10 (-65.27, 77.47)
EtFOSAA	16.03 (-45.66, 77.73)	-28.87 (-94.41, 36.68)	-1.25 (-69.33, 66.82)	-26.27 (-93.09, 40.54)
PFAS mixture [b]	-27.37 (-122.58, 67.85)	-28.39 (-124.50, 67.71)	-3.11 (-99.54, 93.32)	-42.05 (-140.15, 56.04)

Abbrev. PFOA, perfluorooctanoic acid; PFOS, perfluorooctane sulfonic acid; PFHxS, perfluorohexane sulfonic acid; PFNA, perfluorononanoate; MeFOSAA, 2-(N-methyl-perfluorooctane sulfonamido) acetate; EtFOSAA, 2-(N-ethyl-perfluorooctane sulfonamido) acetate.

[a]: Models were adjusted for maternal age (continuous), education (≥ college graduate vs. not college graduate), race and ethnicity (White, Black, Hispanic, Asian, Other), BMI (continuous), smoking history (never, former, early pregnancy smoker), nulliparous (yes vs. no), breastfeeding history (yes vs. no), paternal education (≥ college graduate vs. not college graduate), annual household income (> vs. ≤ $70,000), infertility (yes vs. no), planned pregnancy (yes vs. no), infant sex (male vs. female), gestational age at recruitment (continuous), and AHEI-P score in early pregnancy (continuous).

[b]: Mixture results were obtained from quantile-based g-computation models with single-value imputation (mean for continuous covariates, mode for categorical/binary covariates) for the missingness in covariates.

© 2023 Zhang Y et al. *JAMA Network Open.*

eTable 5. Differences (95% CI) in birthweight z score per doubling of early pregnancy plasma concentrations of per- and polyfluoroalkyl substances across quartile groups by early pregnancy dietary folate intake or plasma folate concentrations among mother-singleton pairs in Project Viva.

	Early Pregnancy Dietary Folate Intake			
	1st Quartile	2nd Quartile	3rd Quartile	4th Quartile
Chemical	Beta (95%CI) [a]	Beta (95%CI) [a]	Beta (95%CI) [a]	Beta (95%CI) [a]
PFOA	**-0.13 (-0.26, -0.003)**	-0.02 (-0.16, 0.13)	-0.04 (-0.17, 0.10)	-0.03 (-0.16, 0.10)
PFOS	-0.04 (-0.15, 0.08)	-0.06 (-0.20, 0.07)	-0.11 (-0.25, 0.02)	-0.06 (-0.20, 0.08)
PFHxS	0.04 (-0.05, 0.12)	-0.03 (-0.13, 0.06)	0.01 (-0.07, 0.09)	-0.02 (-0.11, 0.07)
PFNA	-0.02 (-0.13, 0.10)	-0.09 (-0.22, 0.03)	-0.11 (-0.24, 0.01)	-0.10 (-0.23, 0.03)
MeFOSAA	0.01 (-0.09, 0.11)	-0.02 (-0.12, 0.08)	-0.02 (-0.13, 0.09)	-0.08 (-0.18, 0.02)
EtFOSAA	0.05 (-0.04, 0.14)	-0.04 (-0.12, 0.05)	-0.02 (-0.11, 0.07)	-0.02 (-0.11, 0.07)
PFAS mixture [b]	0.06 (-0.08, 0.19)	-0.07 (-0.20, 0.05)	-0.05 (-0.18, 0.09)	-0.06 (-0.19, 0.08)
	**Early Pregnancy Plasma Folate Concentrations**			
	1st Quartile	2nd Quartile	3rd Quartile	4th Quartile
Chemical	Beta (95%CI) [a]	Beta (95%CI) [a]	Beta (95%CI) [a]	Beta (95%CI) [a]
PFOA	**-0.14 (-0.30, 0.02)**	0.02 (-0.15, 0.19)	-0.05 (-0.23, 0.13)	-0.07 (-0.24, 0.11)
PFOS	-0.11 (-0.28, 0.05)	-0.09 (-0.25, 0.08)	0.03 (-0.15, 0.21)	-0.10 (-0.28, 0.08)
PFHxS	0.03 (-0.08, 0.15)	-0.08 (-0.19, 0.02)	0.04 (-0.06, 0.15)	-0.06 (-0.17, 0.06)
PFNA	-0.08 (-0.25, 0.08)	-0.10 (-0.27, 0.08)	0.05 (-0.12, 0.22)	-0.13 (-0.28, 0.01)
MeFOSAA	-0.09 (-0.23, 0.04)	0.02 (-0.11, 0.15)	-0.01 (-0.14, 0.12)	-0.02 (-0.14, 0.10)
EtFOSAA	0.04 (-0.07, 0.14)	-0.04 (-0.15, 0.08)	0.002 (-0.12, 0.11)	-0.06 (-0.18, 0.05)
PFAS mixture [b]	0.01 (-0.16, 0.17)	-0.03 (-0.19, 0.13)	-0.01 (-0.17, 0.16)	-0.11 (-0.28, 0.06)

Abbrev. PFOA, perfluorooctanoic acid; PFOS, perfluorooctane sulfonic acid; PFHxS, perfluorohexane sulfonic acid; PFNA, perfluorononanoate; MeFOSAA, 2-(N-methyl-perfluorooctane sulfonamido) acetate; EtFOSAA, 2-(N-ethyl-perfluorooctane sulfonamido) acetate.

[a]: Models were adjusted for maternal age (continuous), education (≥ college graduate vs. not college graduate), race and ethnicity (White, Black, Hispanic, Asian, Other), BMI (continuous), smoking history (never, former, early pregnancy smoker), nulliparous (yes vs. no), breastfeeding history (yes vs. no), paternal education (≥ college graduate vs. not college graduate), annual household income (> vs. ≤ $70,000), infertility (yes vs. no), planned pregnancy (yes vs. no), infant sex (male vs. female), gestational age at recruitment (continuous), and AHEI-P score in early pregnancy (continuous).

[b]: Mixture results were obtained from quantile-based g-computation models with single-value imputation (mean for continuous covariates, mode for categorical/binary covariates) for the missingness in covariates.

© 2023 Zhang Y et al. *JAMA Network Open.*

eTable 6. Differences (95% CI) in gestational age (days) per doubling of early pregnancy plasma concentrations of per- and polyfluoroalkyl substances across quartile groups by early pregnancy dietary folate intake or plasma folate concentrations among mother-singleton pairs in Project Viva.

	Early Pregnancy Dietary Folate Intake			
	1st Quartile	2nd Quartile	3rd Quartile	4th Quartile
Chemical	Beta (95%CI) [a]	Beta (95%CI) [a]	Beta (95%CI) [a]	Beta (95%CI) [a]
PFOA	-1.31 (-3.12, 0.51)	-0.91 (-2.91, 1.10)	-0.61 (-2.51, 1.29)	0.41 (-1.42, 2.25)
PFOS	-1.46 (-3.09, 0.17)	-1.28 (-3.16, 0.60)	-0.94 (-2.81, 0.92)	-0.74 (-2.71, 1.23)
PFHxS	-0.96 (-2.20, 0.28)	-0.47 (-1.83, 0.88)	0.13 (-0.99, 1.25)	0.63 (-0.63, 1.89)
PFNA	-0.91 (-2.54, 0.72)	-0.11 (-1.88, 1.66)	0.31 (-1.44, 2.07)	-0.02 (-1.85, 1.80)
MeFOSAA	-0.01 (-1.43, 1.41)	-0.04 (-1.37, 1.30)	0.47 (-1.04, 1.98)	-0.78 (-2.18, 0.62)
EtFOSAA	0.31 (-0.94, 1.57)	-0.03 (-1.21, 1.15)	-0.49 (-1.70, 0.73)	-0.67 (-1.97, 0.62)
PFAS mixture [b]	-0.24 (-2.12, 1.64)	-0.57 (-2.36, 1.22)	-0.13 (-1.99, 1.73)	-0.66 (-2.49, 1.17)
	Early Pregnancy Plasma Folate Concentrations			
	1st Quartile	2nd Quartile	3rd Quartile	4th Quartile
Chemical	Beta (95%CI) [a]	Beta (95%CI) [a]	Beta (95%CI) [a]	Beta (95%CI) [a]
PFOA	-0.98 (-3.12, 1.17)	-0.09 (-2.42, 2.25)	-1.50 (-3.97, 0.97)	0 (-2.38, 2.38)
PFOS	-2.02 (-4.30, 0.26)	-0.42 (-2.66, 1.81)	-0.93 (-3.37, 1.51)	-0.78 (-3.21, 1.65)
PFHxS	0.08 (-1.46, 1.62)	0.05 (-1.35, 1.44)	0.48 (-0.98, 1.93)	-0.36 (-1.94, 1.22)
PFNA	**-2.28 (-4.48, -0.08)**	0.69 (-1.70, 3.08)	1.02 (-1.30, 3.34)	-0.03 (-2.00, 1.94)
MeFOSAA	-1.08 (-2.94, 0.78)	-0.76 (-2.55, 1.04)	-0.82 (-2.59, 0.95)	0.39 (-1.26, 2.03)
EtFOSAA	-0.08 (-1.50, 1.34)	-0.64 (-2.15, 0.87)	-0.20 (-1.77, 1.37)	0.32 (-1.22, 1.86)
PFAS mixture [b]	-1.07 (-3.27, 1.12)	-0.39 (-2.60, 1.83)	0.12 (-2.11, 2.34)	0.27 (-1.99, 2.53)

Abbrev. PFOA, perfluorooctanoic acid; PFOS, perfluorooctane sulfonic acid; PFHxS, perfluorohexane sulfonic acid; PFNA, perfluorononanoate; MeFOSAA, 2-(N-methyl-perfluorooctane sulfonamido) acetate; EtFOSAA, 2-(N-ethyl-perfluorooctane sulfonamido) acetate.

[a]: Models were adjusted for maternal age (continuous), education (≥ college graduatevs. not college graduate), race and ethnicity (White, Black, Hispanic, Asian, Other), BMI (continuous), smoking history (never, former, early pregnancy smoker), nulliparous (yes vs. no), breastfeeding history (yes vs. no), paternal education (≥ college graduate vs. not college graduate), annual household income (> vs. ≤ $70,000), infertility (yes vs. no), planned pregnancy (yes vs. no), infant sex (male vs. female), gestational age at recruitment (continuous), and AHEI-P score in early pregnancy (continuous).

[b]: Mixture results were obtained from quantile-based g-computation models with single-value imputation (mean for continuous covariates, mode for categorical/binary covariates) for the missingness in covariates.

© 2023 Zhang Y et al. *JAMA Network Open.*

eTable 7. Odds Ratios (95% CI) for low birthweight per doubling of early pregnancy plasma concentrations of per- and polyfluoroalkyl substances across quartile groups by early pregnancy dietary folate intake or plasma folate concentrations among mother-singleton pairs in Project Viva.

	Early Pregnancy Dietary Folate Intake	
	1st Quartile	2nd – 4th Quartile
Chemical	OR (95%CI) [a]	OR (95%CI) [a]
PFOA	1.53 (0.77, 3.02)	1.11 (0.69, 1.76)
PFOS	1.60 (0.83, 3.08)	1.41 (0.91, 2.18)
PFHxS	1.21 (0.78, 1.88)	1.00 (0.75, 1.32)
PFNA	1.20 (0.67, 2.12)	1.41 (0.90, 2.21)
MeFOSAA	0.85 (0.52, 1.38)	1.26 (0.90, 1.76)
EtFOSAA	1.05 (0.67, 1.62)	1.03 (0.77, 1.37)
PFAS mixture [b]	1.07 (0.55, 2.06)	1.18 (0.76, 1.84)
	Early Pregnancy Plasma Folate Concentrations	
	1st Quartile	2nd – 4th Quartile
Chemical	OR (95%CI) [a]	OR (95%CI) [a]
PFOA	1.56 (0.70, 3.47)	1.25 (0.61, 2.57)
PFOS	**2.46 (1.05, 5.8)**	1.15 (0.59, 2.21)
PFHxS	1.32 (0.79, 2.18)	0.94 (0.61, 1.44)
PFNA	1.32 (0.64, 2.71)	0.99 (0.54, 1.84)
MeFOSAA	0.86 (0.44, 1.69)	1.43 (0.88, 2.34)
EtFOSAA	1.09 (0.66, 1.81)	0.96 (0.60, 1.52)
PFAS mixture [b]	1.45 (0.63, 3.35)	1.05 (0.53, 2.06)

Abbrev. PFOA, perfluorooctanoic acid; PFOS, perfluorooctane sulfonic acid; PFHxS, perfluorohexane sulfonic acid; PFNA, perfluorononanoate; MeFOSAA, 2-(N-methyl-perfluorooctane sulfonamido) acetate; EtFOSAA, 2-(N-ethyl-perfluorooctane sulfonamido) acetate.

[a]: Models were adjusted for maternal age (continuous), education (≥ college graduate vs. not college graduate), race and ethnicity (White, Black, Hispanic, Asian, Other), BMI (continuous), smoking history (never, former, early pregnancy smoker), nulliparous (yes vs. no), breastfeeding history (yes vs. no), paternal education (≥ college graduate vs. not college graduate), annual household income (> vs. ≤ $70,000), infertility (yes vs. no), planned pregnancy (yes vs. no), infant sex (male vs. female), gestational age at recruitment (continuous), and AHEI-P score in early pregnancy (continuous).

[b]: Mixture results were obtained from quantile-based g-computation models with single-value imputation (mean for continuous covariates, mode for categorical/binary covariates) for the missingness in covariates.

© 2023 Zhang Y et al. *JAMA Network Open.*

eTable 8. Odds Ratios (95% CI) for preterm birth per doubling of early pregnancy plasma concentrations of per- and polyfluoroalkyl substances across quartile groups by early pregnancy dietary folate intake or plasma folate concentrations among mother-singleton pairs in Project Viva.

	Early Pregnancy Dietary Folate Intake	
	1st Quartile	2nd – 4th Quartile
Chemical	OR (95%CI) [a]	OR (95%CI) [a]
PFOA	1.31 (0.77, 2.24)	1.01 (0.69, 1.48)
PFOS	1.55 (0.92, 2.62)	1.14 (0.80, 1.64)
PFHxS	1.20 (0.84, 1.69)	1.08 (0.85, 1.37)
PFNA	1.38 (0.85, 2.25)	1.06 (0.75, 1.51)
MeFOSAA	0.91 (0.61, 1.36)	1.11 (0.84, 1.46)
EtFOSAA	1.15 (0.81, 1.64)	1.00 (0.79, 1.27)
PFAS mixture [b]	1.30 (0.77, 2.22)	1.20 (0.84, 1.72)
	Early Pregnancy Plasma Folate Concentrations	
	1st Quartile	2nd – 4th Quartile
Chemical	OR (95%CI) [a]	OR (95%CI) [a]
PFOA	1.11 (0.60, 2.04)	1.15 (0.66, 2.03)
PFOS	1.51 (0.80, 2.85)	1.16 (0.69, 1.95)
PFHxS	1.03 (0.67, 1.56)	0.95 (0.69, 1.30)
PFNA	1.39 (0.75, 2.57)	0.87 (0.54, 1.41)
MeFOSAA	1.44 (0.83, 2.49)	1.19 (0.80, 1.76)
EtFOSAA	1.14 (0.77, 1.69)	0.99 (0.69, 1.41)
PFAS mixture [b]	1.69 (0.88, 3.25)	0.99 (0.59, 1.66)

Abbrev. PFOA, perfluorooctanoic acid; PFOS, perfluorooctane sulfonic acid; PFHxS, perfluorohexane sulfonic acid; PFNA, perfluorononanoate; MeFOSAA, 2-(N-methyl-perfluorooctane sulfonamido) acetate; EtFOSAA, 2-(N-ethyl-perfluorooctane sulfonamido) acetate.

[a]: Models were adjusted for maternal age (continuous), education (≥ college graduate vs. not college graduate), race and ethnicity (White, Black, Hispanic, Asian, Other), BMI (continuous), smoking history (never, former, early pregnancy smoker), nulliparous (yes vs. no), breastfeeding history (yes vs. no), paternal education (≥ college graduate vs. not college graduate), annual household income (> vs. ≤ $70,000), infertility (yes vs. no), planned pregnancy (yes vs. no), infant sex (male vs. female), gestational age at recruitment (continuous), and AHEI-P score in early pregnancy (continuous).

[b]: Mixture results were obtained from quantile-based g-computation models with single-value imputation (mean for continuous covariates, mode for categorical/binary covariates) for the missingness in covariates.

© 2023 Zhang Y et al. *JAMA Network Open.*

eTable 9. Associations between early pregnancy plasma concentrations of per- and polyfluoroalkyl substances and birth outcomes across quartile groups by early pregnancy dietary folate intake in the subpopulation.

	Early Pregnancy Dietary Folate Intake			
	1st Quartile	2nd Quartile	3rd Quartile	4th Quartile
Dietary folate intake range, mcg/day	128.67 - 676.10	676.10 - 941.94	941.94 - 1194.34	1194.34 - 2874.29
	N=208	N=208	N=207	N=208
	Beta (95%CI) [a]	Beta (95%CI) [a]	Beta (95%CI) [a]	Beta (95%CI) [a]
Birthweight (grams)				
PFOA	**-120.64 (-218.80, -22.48)**	-55.23 (-163.93, 53.46)	6.76 (-95.79, 109.31)	4.03 (-92.13, 100.19)
PFOS	-65.35 (-171.34, 40.65)	-94.25 (-193.24, 4.74)	-25.03 (-128.19, 78.14)	-39.04 (-139.47, 61.39)
PFHxS	5.71 (-61.40, 72.82)	-50.38 (-123.79, 23.03)	-3.44 (-60.04, 53.16)	-6.48 (-71.66, 58.70)
PFNA	-31.24 (-129.54, 67.05)	-67.84 (-159.81, 24.12)	14.70 (-83.41, 112.80)	-60.67 (-158.52, 37.17)
MeFOSAA	0.77 (-74.42, 75.96)	5.07 (-69.37, 79.50)	-17.72 (-101.03, 65.60)	-65.15 (-143.01, 12.72)
EtFOSAA	24.58 (-43.11, 92.27)	-19.71 (-82.60, 43.18)	-6.63 (-72.02, 58.75)	-34.66 (-103.56, 34.24)
Birthweight Z-score				
PFOA	**-0.17 (-0.34, -0.003)**	-0.11 (-0.30, 0.07)	0.04 (-0.14, 0.21)	0.01 (-0.15, 0.17)
PFOS	-0.13 (-0.31, 0.05)	-0.15 (-0.32, 0.02)	0 (-0.18, 0.17)	0 (-0.17, 0.17)
PFHxS	0.01 (-0.10, 0.12)	-0.06 (-0.18, 0.07)	0 (-0.10, 0.10)	-0.04 (-0.15, 0.07)
PFNA	-0.08 (-0.24, 0.09)	-0.10 (-0.26, 0.05)	-0.01 (-0.18, 0.16)	-0.10 (-0.26, 0.07)
MeFOSAA	0.04 (-0.09, 0.16)	-0.01 (-0.14, 0.11)	-0.03 (-0.17, 0.11)	-0.06 (-0.2, 0.07)
EtFOSAA	0.03 (-0.08, 0.14)	-0.07 (-0.18, 0.03)	0.02 (-0.09, 0.13)	-0.02 (-0.14, 0.09)
Gestational Age (days)				
PFOA	-2.06 (-4.31, 0.20)	-0.28 (-2.78, 2.21)	-0.51 (-2.86, 1.85)	0.33 (-1.88, 2.54)
PFOS	-0.76 (-3.19, 1.68)	-1.27 (-3.54, 1)	-1.21 (-3.58, 1.15)	-1.37 (-3.67, 0.94)
PFHxS	-0.27 (-1.81, 1.27)	-0.79 (-2.48, 0.89)	0.36 (-0.94, 1.66)	0.65 (-0.84, 2.15)
PFNA	-0.28 (-2.54, 1.97)	-0.67 (-2.78, 1.45)	0.52 (-1.73, 2.78)	-0.56 (-2.80, 1.69)
MeFOSAA	-1.11 (-2.83, 0.61)	0.72 (-0.98, 2.43)	-0.10 (-2.01, 1.80)	-1.54 (-3.33, 0.24)
EtFOSAA	0.47 (-1.08, 2.02)	0.51 (-0.93, 1.95)	-0.62 (-2.12, 0.88)	-1.18 (-2.75, 0.40)
	1st Quartile	2nd - 4th Quartile		
	OR (95%CI) [a]	OR (95%CI) [a]		
Preterm Birth				
PFOA	1.85 (0.80, 4.29)	0.97 (0.59, 1.61)		
PFOS	1.74 (0.78, 3.87)	1.22 (0.76, 1.95)		
PFHxS	0.85 (0.50, 1.43)	1.06 (0.79, 1.43)		
PFNA	1.09 (0.55, 2.18)	1.04 (0.66, 1.63)		
MeFOSAA	1.19 (0.66, 2.12)	1.29 (0.89, 1.87)		
EtFOSAA	1.19 (0.70, 2.01)	1.01 (0.74, 1.38)		
Low Birthweight				
PFOA	1.62 (0.63, 4.15)	1.28 (0.65, 2.52)		

© 2023 Zhang Y et al. *JAMA Network Open.*

PFOS	1.49 (0.58, 3.85)	1.60 (0.86, 2.99)
PFHxS	0.89 (0.47, 1.69)	1.20 (0.80, 1.78)
PFNA	0.80 (0.38, 1.71)	1.38 (0.74, 2.56)
MeFOSAA	0.82 (0.43, 1.55)	1.47 (0.90, 2.40)
EtFOSAA	1.17 (0.65, 2.13)	0.94 (0.61, 1.44)

Abbrev. PFOA, perfluorooctanoic acid; PFOS, perfluorooctane sulfonic acid; PFHxS, perfluorohexane sulfonic acid; PFNA, perfluorononanoate; MeFOSAA, 2-(N-methyl-perfluorooctane sulfonamido) acetate; EtFOSAA, 2-(N-ethyl-perfluorooctane sulfonamido) acetate.

[a]: Models were adjusted for maternal age (continuous), education (≥ college graduate vs. not college graduate), race and ethnicity (White, Black, Hispanic, Asian, Other), BMI (continuous), smoking history (never, former, early pregnancy smoker), nulliparous (yes vs. no), breastfeeding history (yes vs. no), paternal education (≥ college graduate vs. not college graduate), annual household income (> vs. ≤ $70,000), infertility (yes vs. no), planned pregnancy (yes vs. no), infant sex (male vs. female), gestational age at recruitment (continuous), and AHEI-P score in early pregnancy (continuous).

© 2023 Zhang Y et al. *JAMA Network Open.*

eTable 10. Associations between early pregnancy plasma concentrations of per- and polyfluoroalkyl substances and birth outcomes across quartile groups by early pregnancy dietary folate intake, further adjusting for average fish consumption in early pregnancy.

		Early Pregnancy Dietary Folate Intake			
		1st Quartile Beta (95%CI) [a]	2nd Quartile Beta (95%CI) [a]	3rd Quartile Beta (95%CI) [a]	4th Quartile Beta (95%CI) [a]
Birthweight (grams)					
	PFOA	**-84.72 (-162.66, -6.79)**	-29.82 (-115.7, 56.06)	-28.77 (-110.21, 52.66)	-6.72 (-85.29, 71.85)
	PFOS	-33.78 (-103.54, 35.98)	-51.22 (-131.98, 29.53)	-71.66 (-151.64, 8.33)	-55.97 (-140.24, 28.31)
	PFHxS	-0.62 (-53.65, 52.42)	-30.82 (-88.83, 27.19)	3.57 (-44.60, 51.73)	5.30 (-48.64, 59.23)
	PFNA	-23.04 (-93.14, 47.06)	-50.15 (-126.39, 26.09)	-41.34 (-116.63, 33.96)	-52.20 (-130.28, 25.88)
	MeFOSAA	11.41 (-49.47, 72.3)	-13.55 (-70.67, 43.57)	-1.31 (-65.90, 63.28)	-55.20 (-114.98, 4.58)
	EtFOSAA	29.37 (-24.18, 82.92)	-14.35 (-64.89, 36.18)	-24.10 (-76.13, 27.93)	-22.27 (-77.81, 33.28)
Birthweight Z-score					
	PFOA	-0.12 (-0.25, 0.01)	-0.01 (-0.16, 0.13)	-0.03 (-0.17, 0.11)	-0.03 (-0.16, 0.10)
	PFOS	-0.03 (-0.15, 0.09)	-0.05 (-0.19, 0.08)	-0.11 (-0.24, 0.03)	-0.06 (-0.20, 0.08)
	PFHxS	0.04 (-0.05, 0.12)	-0.03 (-0.13, 0.06)	0.02 (-0.06, 0.10)	-0.01 (-0.10, 0.08)
	PFNA	-0.01 (-0.12, 0.11)	-0.08 (-0.21, 0.04)	-0.11 (-0.23, 0.02)	-0.09 (-0.22, 0.04)
	MeFOSAA	0.01 (-0.09, 0.11)	-0.02 (-0.11, 0.08)	-0.02 (-0.13, 0.09)	-0.08 (-0.18, 0.02)
	EtFOSAA	0.05 (-0.04, 0.14)	-0.03 (-0.12, 0.05)	-0.02 (-0.11, 0.07)	-0.02 (-0.11, 0.08)
Gestational Age (days)					
	PFOA	-1.30 (-3.12, 0.52)	-0.89 (-2.90, 1.11)	-0.59 (-2.49, 1.31)	0.41 (-1.43, 2.25)
	PFOS	-1.46 (-3.09, 0.17)	-1.25 (-3.14, 0.64)	-0.94 (-2.81, 0.93)	-0.75 (-2.72, 1.22)
	PFHxS	-0.97 (-2.21, 0.27)	-0.46 (-1.82, 0.89)	0.14 (-0.99, 1.26)	0.63 (-0.63, 1.89)
	PFNA	-0.92 (-2.56, 0.72)	-0.07 (-1.85, 1.71)	0.31 (-1.45, 2.07)	-0.03 (-1.85, 1.8)
	MeFOSAA	0 (-1.43, 1.42)	-0.02 (-1.36, 1.31)	0.47 (-1.04, 1.98)	-0.80 (-2.20, 0.60)
	EtFOSAA	0.32 (-0.93, 1.57)	-0.02 (-1.20, 1.17)	-0.48 (-1.69, 0.74)	-0.67 (-1.97, 0.63)
		1st Quartile OR (95%CI) [a]	2nd - 4th Quartile OR (95%CI) [a]		
Preterm Birth					
	PFOA	1.31 (0.77, 2.23)	1 (0.68, 1.47)		
	PFOS	1.56 (0.93, 2.61)	1.15 (0.80, 1.65)		
	PFHxS	1.20 (0.84, 1.70)	1.08 (0.86, 1.37)		
	PFNA	1.43 (0.88, 2.33)	1.09 (0.76, 1.55)		
	MeFOSAA	0.90 (0.60, 1.33)	1.11 (0.84, 1.46)		
	EtFOSAA	1.14 (0.80, 1.62)	1 (0.79, 1.27)		
Low Birthweight					
	PFOA	1.55 (0.78, 3.05)	1.11 (0.70, 1.77)		
	PFOS	1.62 (0.84, 3.10)	1.42 (0.91, 2.21)		
	PFHxS	1.22 (0.79, 1.89)	1 (0.75, 1.33)		
	PFNA	1.24 (0.69, 2.21)	1.44 (0.91, 2.26)		

© 2023 Zhang Y et al. *JAMA Network Open.*

MeFOSAA	0.85 (0.53, 1.38)	1.26 (0.90, 1.77)
EtFOSAA	1.04 (0.67, 1.61)	1.03 (0.77, 1.37)

Abbrev. PFOA, perfluorooctanoic acid; PFOS, perfluorooctane sulfonic acid; PFHxS, perfluorohexane sulfonic acid; PFNA, perfluorononanoate; MeFOSAA, 2-(N-methyl-perfluorooctane sulfonamido) acetate; EtFOSAA, 2-(N-ethyl-perfluorooctane sulfonamido) acetate.

[a]: Models were adjusted for maternal age (continuous), education (≥ college graduate vs. not college graduate), race and ethnicity (White, Black, Hispanic, Asian, Other), BMI (continuous), smoking history (never, former, early pregnancy smoker), nulliparous (yes vs. no), breastfeeding history (yes vs. no), paternal education (≥ college graduate vs. not college graduate), annual household income (> vs. ≤ $70,000), infertility (yes vs. no), planned pregnancy (yes vs. no), infant sex (male vs. female), gestational age at recruitment (continuous), AHEI-P score in early pregnancy (continuous), and average fish consumption in early pregnancy (continuous).

© 2023 Zhang Y et al. *JAMA Network Open.*

eTable 11. Associations between early pregnancy plasma concentrations of per- and polyfluoroalkyl substances and birth outcomes across quartile groups by early pregnancy plasma folate concentrations, further adjusting for average fish consumption in early pregnancy.

		Early Pregnancy Plasma Folate Concentrations			
		1st Quartile<br>Beta (95%CI) [a]	2nd Quartile<br>Beta (95%CI) [a]	3rd Quartile<br>Beta (95%CI) [a]	4th Quartile<br>Beta (95%CI) [a]
Birthweight (grams)					
	PFOA	**-85.25 (-178.43, 7.92)**	6.76 (-94.61, 108.14)	-62.14 (-169.21, 44.94)	-21.70 (-124.96, 81.57)
	PFOS	**-98.83 (-197.86, 0.19)**	-53.42 (-150.54, 43.71)	-1.15 (-107.18, 104.89)	-48.16 (-153.96, 57.63)
	PFHxS	8.81 (-58.05, 75.66)	-43.26 (-103.92, 17.40)	24.81 (-38.40, 88.01)	-27.32 (-96.11, 41.48)
	PFNA	**-91.00 (-186.70, 4.70)**	-26.26 (-130.90, 78.38)	50.80 (-50.06, 151.65)	-56.15 (-141.86, 29.56)
	MeFOSAA	-59.29 (-140.09, 21.5)	-8.40 (-86.48, 69.68)	-33.58 (-110.51, 43.36)	7.22 (-64.25, 78.68)
	EtFOSAA	17.36 (-44.41, 79.13)	-27.76 (-93.36, 37.84)	-1.66 (-69.74, 66.41)	-24.65 (-91.66, 42.35)
Birthweight Z-score					
	PFOA	-0.14 (-0.30, 0.02)	0.03 (-0.14, 0.20)	-0.05 (-0.23, 0.13)	-0.06 (-0.24, 0.11)
	PFOS	-0.11 (-0.28, 0.06)	-0.08 (-0.25, 0.08)	0.03 (-0.15, 0.21)	-0.09 (-0.27, 0.09)
	PFHxS	0.03 (-0.08, 0.15)	-0.08 (-0.19, 0.02)	0.04 (-0.06, 0.15)	-0.05 (-0.17, 0.06)
	PFNA	-0.08 (-0.24, 0.08)	-0.08 (-0.26, 0.09)	0.06 (-0.11, 0.23)	-0.13 (-0.27, 0.02)
	MeFOSAA	-0.09 (-0.23, 0.05)	0.02 (-0.12, 0.15)	-0.01 (-0.14, 0.12)	-0.01 (-0.13, 0.11)
	EtFOSAA	0.04 (-0.06, 0.15)	-0.03 (-0.14, 0.08)	0 (-0.12, 0.11)	-0.06 (-0.17, 0.05)
Gestational Age (days)					
	PFOA	-0.99 (-3.14, 1.16)	-0.11 (-2.45, 2.23)	-1.51 (-3.98, 0.96)	-0.02 (-2.41, 2.36)
	PFOS	-2.06 (-4.34, 0.22)	-0.47 (-2.71, 1.77)	-0.95 (-3.40, 1.49)	-0.84 (-3.28, 1.60)
	PFHxS	0.08 (-1.47, 1.62)	0.05 (-1.35, 1.45)	0.47 (-0.98, 1.93)	-0.39 (-1.98, 1.20)
	PFNA	**-2.33 (-4.54, -0.13)**	0.62 (-1.79, 3.03)	0.99 (-1.33, 3.32)	-0.07 (-2.05, 1.90)
	MeFOSAA	-1.09 (-2.95, 0.77)	-0.73 (-2.53, 1.07)	-0.82 (-2.59, 0.96)	0.37 (-1.28, 2.01)
	EtFOSAA	-0.08 (-1.50, 1.35)	-0.64 (-2.16, 0.87)	-0.19 (-1.76, 1.37)	0.29 (-1.25, 1.84)
		1st Quartile<br>OR (95%CI) [a]	2nd - 4th Quartile<br>OR (95%CI) [a]		
Preterm Birth					
	PFOA	1.11 (0.60, 2.06)	1.18 (0.66, 2.08)		
	PFOS	1.53 (0.81, 2.90)	1.21 (0.71, 2.07)		
	PFHxS	1.02 (0.67, 1.56)	0.96 (0.69, 1.32)		
	PFNA	1.43 (0.77, 2.64)	0.90 (0.55, 1.47)		
	MeFOSAA	1.44 (0.83, 2.50)	1.19 (0.80, 1.77)		
	EtFOSAA	1.13 (0.76, 1.68)	1 (0.70, 1.43)		
Low Birthweight					
	PFOA	1.58 (0.71, 3.54)	1.32 (0.63, 2.75)		
	PFOS	**2.55 (1.08, 6.03)**	1.25 (0.63, 2.47)		
	PFHxS	1.33 (0.80, 2.23)	0.97 (0.63, 1.5)		

© 2023 Zhang Y et al. *JAMA Network Open.*

PFNA	1.35 (0.66, 2.75)	1.05 (0.56, 1.97)
MeFOSAA	0.87 (0.44, 1.7)	1.44 (0.87, 2.38)
EtFOSAA	1.10 (0.66, 1.82)	0.99 (0.62, 1.58)

Abbrev. PFOA, perfluorooctanoic acid; PFOS, perfluorooctane sulfonic acid; PFHxS, perfluorohexane sulfonic acid; PFNA, perfluorononanoate; MeFOSAA, 2-(N-methyl-perfluorooctane sulfonamido) acetate; EtFOSAA, 2-(N-ethyl-perfluorooctane sulfonamido) acetate.
[a]: Models were adjusted for maternal age (continuous), education (≥ college graduate vs. not college graduate), race and ethnicity (White, Black, Hispanic, Asian, Other), BMI (continuous), smoking history (never, former, early pregnancy smoker), nulliparous (yes vs. no), breastfeeding history (yes vs. no), paternal education (≥ college graduate vs. not college graduate), annual household income (> vs. ≤ $70,000), infertility (yes vs. no), planned pregnancy (yes vs. no), infant sex (male vs. female), gestational age at recruitment (continuous), AHEI-P score in early pregnancy (continuous), and average fish consumption in early pregnancy (continuous).

© 2023 Zhang Y et al. *JAMA Network Open.*

eTable 12. Differences (95% CI) in birthweight (grams) per doubling of early pregnancy plasma concentrations of per- and polyfluoroalkyl substances across quartile groups by early pregnancy dietary folate intake or plasma folate concentrations among mother-singleton pairs in Project Viva, using single-value imputation to account for missingness.

	Early Pregnancy Dietary Folate Intake			
	1st Quartile	2nd Quartile	3rd Quartile	4th Quartile
Chemical	Beta (95%CI) [a]	Beta (95%CI) [a]	Beta (95%CI) [a]	Beta (95%CI) [a]
PFOA	**-88.41 (-165.94, -10.88)**	-30.78 (-116.67, 55.11)	-31.44 (-112.66, 49.78)	-1.58 (-80.53, 77.37)
PFOS	-36.76 (-106.39, 32.86)	-57.95 (-138.38, 22.48)	-73.62 (-153.49, 6.26)	-53.25 (-137.74, 31.24)
PFHxS	0.84 (-52.17, 53.85)	-30.77 (-88.83, 27.30)	1.32 (-46.79, 49.43)	8.69 (-45.55, 62.93)
PFNA	-25.08 (-94.66, 44.50)	-56.36 (-132.08, 19.35)	-43.12 (-118.29, 32.06)	-50.83 (-129.12, 27.46)
MeFOSAA	11.96 (-48.87, 72.79)	-16.28 (-73.38, 40.82)	-1.37 (-66.04, 63.30)	-51.53 (-111.66, 8.60)
EtFOSAA	28.08 (-25.47, 81.63)	-16.25 (-66.81, 34.30)	-24.3 (-76.36, 27.77)	-21.28 (-77.11, 34.54)
	Early Pregnancy Plasma Folate Concentrations			
	1st Quartile	2nd Quartile	3rd Quartile	4th Quartile
Chemical	Beta (95%CI) [a]	Beta (95%CI) [a]	Beta (95%CI) [a]	Beta (95%CI) [a]
PFOA	**-88.53 (-181.46, 4.39)**	4.71 (-96.66, 106.08)	-63.37 (-170.43, 43.69)	-23.37 (-126.58, 79.84)
PFOS	**-100.57 (-199.43, -1.72)**	-55.79 (-152.68, 41.11)	-0.33 (-106.28, 105.62)	-48.88 (-154.36, 56.60)
PFHxS	9.70 (-57.14, 76.53)	-43.79 (-104.46, 16.88)	24.36 (-38.87, 87.58)	-27.59 (-96.21, 41.02)
PFNA	**-88.65 (-184.08, 6.77)**	-30.26 (-134.12, 73.59)	49.81 (-50.86, 150.48)	-57.06 (-142.57, 28.45)
MeFOSAA	-60.51 (-141.29, 20.27)	-7.73 (-85.78, 70.33)	-30.23 (-107.06, 46.60)	5.58 (-65.78, 76.95)
EtFOSAA	14.27 (-47.36, 75.91)	-28.16 (-93.80, 37.47)	-0.94 (-69.03, 67.15)	-27.08 (-94.01, 39.84)

Abbrev. PFOA, perfluorooctanoic acid; PFOS, perfluorooctane sulfonic acid; PFHxS, perfluorohexane sulfonic acid; PFNA, perfluorononanoate; MeFOSAA, 2-(N-methyl-perfluorooctane sulfonamido) acetate; EtFOSAA, 2-(N-ethyl-perfluorooctane sulfonamido) acetate.

[a]: Models were adjusted for maternal age (continuous), education (≥ college graduatevs. not college graduate), race and ethnicity (White, Black, Hispanic, Asian, Other), BMI (continuous), smoking history (never, former, early pregnancy smoker), nulliparous (yes vs. no), breastfeeding history (yes vs. no), paternal education (≥ college graduate vs. not college graduate), annual household income (> vs. ≤ $70,000), infertility (yes vs. no), planned pregnancy (yes vs. no), infant sex (male vs. female), gestational age at recruitment (continuous), and AHEI-P score in early pregnancy (continuous).

© 2023 Zhang Y et al. *JAMA Network Open.*

eTable 13. Differences (95% CI) in birthweight z score per doubling of early pregnancy plasma concentrations of per- and polyfluoroalkyl substances across quartile groups by early pregnancy dietary folate intake or plasma folate concentrations among mother-singleton pairs in Project Viva, using single-value imputation to account for missingness.

	Early Pregnancy Dietary Folate Intake			
	1st Quartile	2nd Quartile	3rd Quartile	4th Quartile
Chemical	Beta (95%CI) [a]	Beta (95%CI) [a]	Beta (95%CI) [a]	Beta (95%CI) [a]
PFOA	**-0.13 (-0.26, -0.005)**	-0.02 (-0.16, 0.12)	-0.04 (-0.17, 0.10)	-0.02 (-0.15, 0.12)
PFOS	-0.04 (-0.16, 0.08)	-0.07 (-0.20, 0.07)	-0.11 (-0.25, 0.02)	-0.06 (-0.20, 0.08)
PFHxS	0.04 (-0.05, 0.13)	-0.03 (-0.13, 0.06)	0.01 (-0.07, 0.09)	-0.01 (-0.10, 0.08)
PFNA	-0.01 (-0.13, 0.10)	-0.10 (-0.22, 0.03)	-0.12 (-0.24, 0.01)	-0.09 (-0.22, 0.04)
MeFOSAA	0.01 (-0.09, 0.11)	-0.02 (-0.12, 0.07)	-0.02 (-0.13, 0.09)	-0.07 (-0.17, 0.03)
EtFOSAA	0.04 (-0.04, 0.13)	-0.04 (-0.12, 0.05)	-0.02 (-0.11, 0.07)	-0.01 (-0.11, 0.08)

	Early Pregnancy Plasma Folate Concentrations			
	1st Quartile	2nd Quartile	3rd Quartile	4th Quartile
Chemical	Beta (95%CI) [a]	Beta (95%CI) [a]	Beta (95%CI) [a]	Beta (95%CI) [a]
PFOA	-0.14 (-0.30, 0.01)	0.02 (-0.15, 0.20)	-0.05 (-0.23, 0.13)	-0.07 (-0.25, 0.10)
PFOS	-0.11 (-0.28, 0.05)	-0.09 (-0.25, 0.08)	0.03 (-0.15, 0.21)	-0.10 (-0.28, 0.08)
PFHxS	0.03 (-0.08, 0.15)	-0.08 (-0.19, 0.02)	0.04 (-0.06, 0.15)	-0.05 (-0.17, 0.06)
PFNA	-0.08 (-0.24, 0.08)	-0.10 (-0.27, 0.08)	0.05 (-0.12, 0.22)	-0.13 (-0.28, 0.01)
MeFOSAA	-0.09 (-0.23, 0.04)	0.02 (-0.11, 0.15)	0 (-0.13, 0.13)	-0.02 (-0.14, 0.10)
EtFOSAA	0.03 (-0.07, 0.14)	-0.03 (-0.15, 0.08)	0 (-0.12, 0.11)	-0.07 (-0.18, 0.05)

Abbrev. PFOA, perfluorooctanoic acid; PFOS, perfluorooctane sulfonic acid; PFHxS, perfluorohexane sulfonic acid; PFNA, perfluorononanoate; MeFOSAA, 2-(N-methyl-perfluorooctane sulfonamido) acetate; EtFOSAA, 2-(N-ethyl-perfluorooctane sulfonamido) acetate.

[a]: Models were adjusted for maternal age (continuous), education (≥ college graduate vs. not college graduate), race and ethnicity (White, Black, Hispanic, Asian, Other), BMI (continuous), smoking history (never, former, early pregnancy smoker), nulliparous (yes vs. no), breastfeeding history (yes vs. no), paternal education (≥ college graduate vs. not college graduate), annual household income (> vs. ≤ $70,000), infertility (yes vs. no), planned pregnancy (yes vs. no), infant sex (male vs. female), gestational age at recruitment (continuous), and AHEI-P score in early pregnancy (continuous).

© 2023 Zhang Y et al. *JAMA Network Open.*

eTable 14. Differences (95% CI) in gestational age (days) per doubling of early pregnancy plasma concentrations of per- and polyfluoroalkyl substances across quartile groups by early pregnancy dietary folate intake or plasma folate concentrations among mother-singleton pairs in Project Viva, using single-value imputation to account for missingness.

	Early Pregnancy Dietary Folate Intake			
	1st Quartile	2nd Quartile	3rd Quartile	4th Quartile
Chemical	Beta (95%CI) [a]	Beta (95%CI) [a]	Beta (95%CI) [a]	Beta (95%CI) [a]
PFOA	-1.26 (-3.07, 0.55)	-0.79 (-2.8, 1.21)	-0.53 (-2.43, 1.37)	0.39 (-1.45, 2.24)
PFOS	-1.42 (-3.04, 0.21)	-1.22 (-3.1, 0.66)	-0.88 (-2.75, 0.98)	-0.76 (-2.73, 1.22)
PFHxS	-0.93 (-2.17, 0.30)	-0.45 (-1.8, 0.9)	0.12 (-1, 1.24)	0.63 (-0.64, 1.9)
PFNA	-0.87 (-2.49, 0.76)	-0.08 (-1.85, 1.69)	0.37 (-1.39, 2.13)	-0.03 (-1.86, 1.8)
MeFOSAA	0.03 (-1.39, 1.45)	-0.05 (-1.38, 1.29)	0.47 (-1.04, 1.98)	-0.81 (-2.22, 0.59)
EtFOSAA	0.33 (-0.92, 1.58)	0 (-1.18, 1.18)	-0.44 (-1.66, 0.78)	-0.71 (-2.02, 0.59)
	Early Pregnancy Plasma Folate Concentrations			
	1st Quartile	2nd Quartile	3rd Quartile	4th Quartile
Chemical	Beta (95%CI) [a]	Beta (95%CI) [a]	Beta (95%CI) [a]	Beta (95%CI) [a]
PFOA	-1.00 (-3.14, 1.14)	-0.07 (-2.41, 2.27)	-1.50 (-3.97, 0.97)	0.02 (-2.36, 2.40)
PFOS	-2.02 (-4.3, 0.26)	-0.41 (-2.65, 1.82)	-0.91 (-3.35, 1.53)	-0.76 (-3.19, 1.67)
PFHxS	0.09 (-1.45, 1.63)	0.05 (-1.34, 1.45)	0.47 (-0.99, 1.92)	-0.35 (-1.94, 1.23)
PFNA	**-2.25 (-4.45, -0.06)**	0.70 (-1.70, 3.09)	1.02 (-1.30, 3.33)	-0.02 (-1.99, 1.95)
MeFOSAA	-1.08 (-2.94, 0.78)	-0.75 (-2.55, 1.04)	-0.79 (-2.56, 0.97)	0.38 (-1.26, 2.03)
EtFOSAA	-0.10 (-1.52, 1.32)	-0.63 (-2.14, 0.89)	-0.19 (-1.76, 1.38)	0.30 (-1.24, 1.85)

Abbrev. PFOA, perfluorooctanoic acid; PFOS, perfluorooctane sulfonic acid; PFHxS, perfluorohexane sulfonic acid; PFNA, perfluorononanoate; MeFOSAA, 2-(N-methyl-perfluorooctane sulfonamido) acetate; EtFOSAA, 2-(N-ethyl-perfluorooctane sulfonamido) acetate.

[a]: Models were adjusted for maternal age (continuous), education (≥ college graduate vs. not college graduate), race and ethnicity (White, Black, Hispanic, Asian, Other), BMI (continuous), smoking history (never, former, early pregnancy smoker), nulliparous (yes vs. no), breastfeeding history (yes vs. no), paternal education (≥ college graduate vs. not college graduate), annual household income (> vs. ≤ $70,000), infertility (yes vs. no), planned pregnancy (yes vs. no), infant sex (male vs. female), gestational age at recruitment (continuous), and AHEI-P score in early pregnancy (continuous).

© 2023 Zhang Y et al. *JAMA Network Open.*

eTable 15. Odds Ratios (95% CI) for low birthweight per doubling of early pregnancy plasma concentrations of per- and polyfluoroalkyl substances across quartile groups by early pregnancy dietary folate intake or plasma folate concentrations among mother-singleton pairs in Project Viva, using single-value imputation to account for missingness.

	Early Pregnancy Dietary Folate Intake	
	1st Quartile	2nd – 4th Quartile
Chemical	OR (95%CI) [a]	OR (95%CI) [a]
PFOA	1.56 (0.78, 3.08)	1.10 (0.69, 1.76)
PFOS	1.60 (0.83, 3.1)	1.41 (0.91, 2.19)
PFHxS	1.22 (0.79, 1.9)	1 (0.75, 1.32)
PFNA	1.18 (0.66, 2.1)	1.39 (0.89, 2.17)
MeFOSAA	0.85 (0.53, 1.38)	1.26 (0.90, 1.77)
EtFOSAA	1.04 (0.67, 1.62)	1.02 (0.77, 1.36)
	Early Pregnancy Plasma Folate Concentrations	
	1st Quartile	2nd – 4th Quartile
Chemical	OR (95%CI) [a]	OR (95%CI) [a]
PFOA	1.65 (0.74, 3.66)	1.28 (0.62, 2.62)
PFOS	**2.57 (1.08, 6.10)**	1.13 (0.59, 2.19)
PFHxS	1.34 (0.81, 2.21)	0.95 (0.62, 1.45)
PFNA	1.34 (0.64, 2.79)	0.99 (0.54, 1.83)
MeFOSAA	0.87 (0.44, 1.71)	1.45 (0.89, 2.37)
EtFOSAA	1.12 (0.68, 1.84)	0.96 (0.60, 1.52)

Abbrev. PFOA, perfluorooctanoic acid; PFOS, perfluorooctane sulfonic acid; PFHxS, perfluorohexane sulfonic acid; PFNA, perfluorononanoate; MeFOSAA, 2-(N-methyl-perfluorooctane sulfonamido) acetate; EtFOSAA, 2-(N-ethyl-perfluorooctane sulfonamido) acetate.

[a]: Models were adjusted for maternal age (continuous), education (≥ college graduate vs. not college graduate), race and ethnicity (White, Black, Hispanic, Asian, Other), BMI (continuous), smoking history (never, former, early pregnancy smoker), nulliparous (yes vs. no), breastfeeding history (yes vs. no), paternal education (≥ college graduate vs. not college graduate), annual household income (> vs. ≤ $70,000), infertility (yes vs. no), planned pregnancy (yes vs. no), infant sex (male vs. female), gestational age at recruitment (continuous), and AHEI-P score in early pregnancy (continuous).

© 2023 Zhang Y et al. *JAMA Network Open.*

eTable 16. Odds Ratios (95% CI) for preterm birth per doubling of early pregnancy plasma concentrations of per- and polyfluoroalkyl substances across quartile groups by early pregnancy dietary folate intake or plasma folate concentrations among mother-singleton pairs in Project Viva, using single-value imputation to account for missingness.

	Early Pregnancy Dietary Folate Intake	
	1st Quartile	2nd – 4th Quartile
Chemical	OR (95%CI) [a]	OR (95%CI) [a]
PFOA	1.30 (0.75, 2.23)	0.99 (0.68, 1.45)
PFOS	1.53 (0.91, 2.58)	1.13 (0.79, 1.61)
PFHxS	1.19 (0.83, 1.69)	1.08 (0.85, 1.36)
PFNA	1.35 (0.83, 2.2)	1.05 (0.74, 1.48)
MeFOSAA	0.90 (0.60, 1.33)	1.11 (0.85, 1.46)
EtFOSAA	1.14 (0.80, 1.62)	1.00 (0.79, 1.26)
	Early Pregnancy Plasma Folate Concentrations	
	1st Quartile	2nd – 4th Quartile
Chemical	OR (95%CI) [a]	OR (95%CI) [a]
PFOA	1.16 (0.63, 2.13)	1.17 (0.66, 2.06)
PFOS	1.54 (0.82, 2.91)	1.16 (0.69, 1.95)
PFHxS	1.04 (0.68, 1.58)	0.95 (0.69, 1.31)
PFNA	1.39 (0.75, 2.58)	0.86 (0.53, 1.40)
MeFOSAA	1.44 (0.84, 2.49)	1.19 (0.81, 1.76)
EtFOSAA	1.16 (0.79, 1.72)	0.99 (0.70, 1.41)

Abbrev. PFOA, perfluorooctanoic acid; PFOS, perfluorooctane sulfonic acid; PFHxS, perfluorohexane sulfonic acid; PFNA, perfluorononanoate; MeFOSAA, 2-(N-methyl-perfluorooctane sulfonamido) acetate; EtFOSAA, 2-(N-ethyl-perfluorooctane sulfonamido) acetate.

[a]: Models were adjusted for maternal age (continuous), education (≥ college graduate vs. not college graduate), race and ethnicity (White, Black, Hispanic, Asian, Other), BMI (continuous), smoking history (never, former, early pregnancy smoker), nulliparous (yes vs. no), breastfeeding history (yes vs. no), paternal education (≥ college graduatevs. not college graduate), annual household income (> vs. ≤ $70,000), infertility (yes vs. no), planned pregnancy (yes vs. no), infant sex (male vs. female), gestational age at recruitment (continuous), and AHEI-P score in early pregnancy (continuous).

© 2023 Zhang Y et al. *JAMA Network Open.*

eFigure 1. Participants' inclusion diagram of mother-singleton pairs in Project Viva.

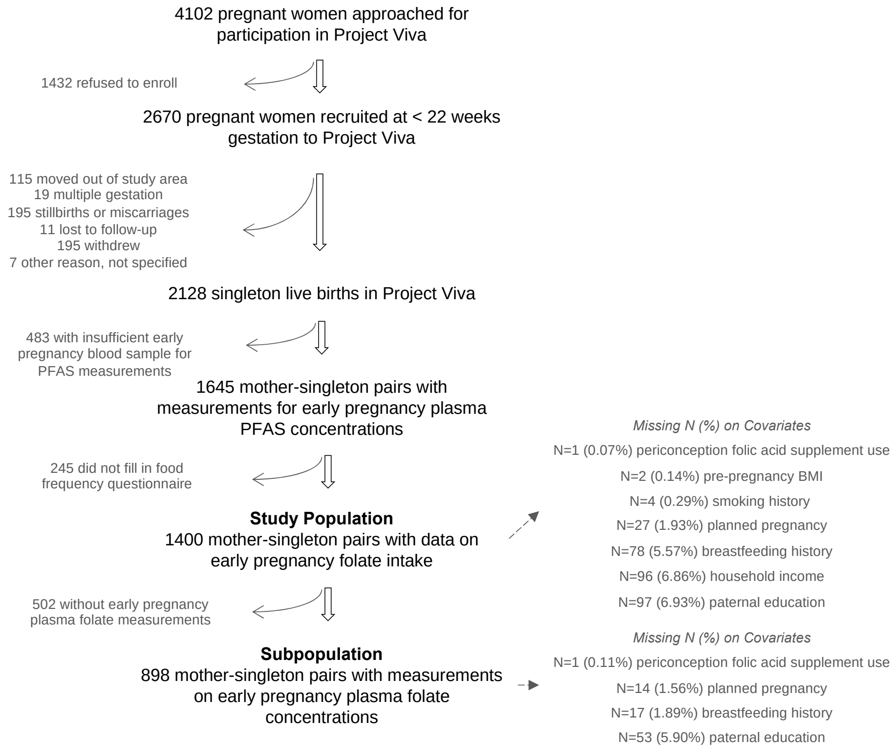

Abbrev. PFAS, per- and polyfluoroalkyl substances; BMI, body mass index.

© 2023 Zhang Y et al. *JAMA Network Open.*

eFigure 2. Directed Acyclic Graph under the null hypothesis of PFAS-birth outcome associations.

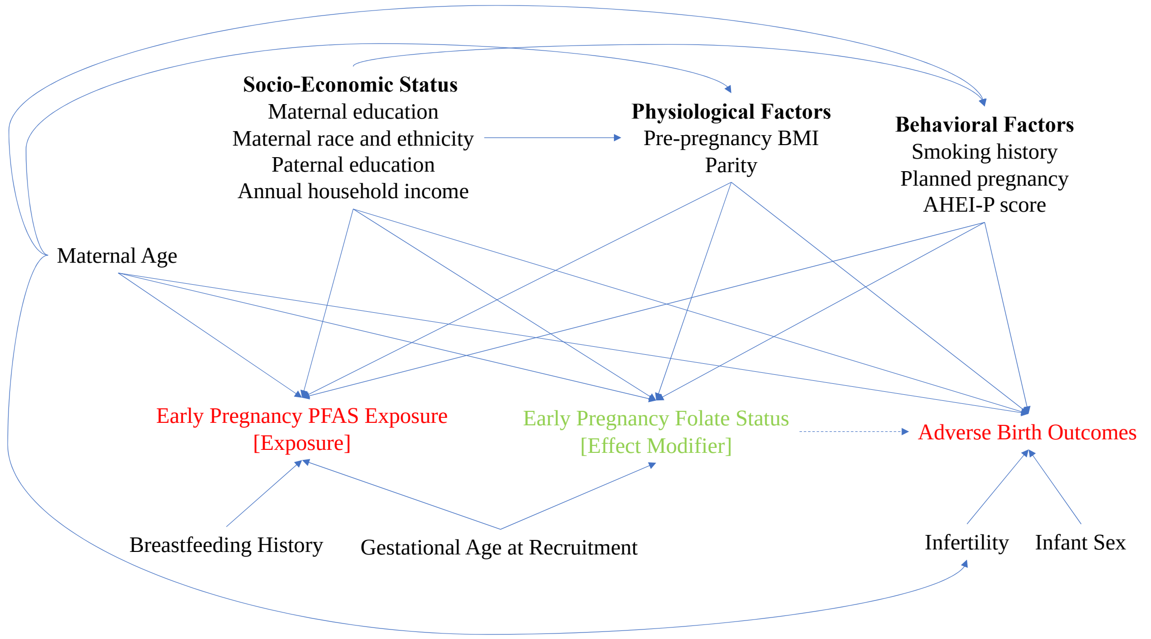

Abbrev. PFAS, per- and polyfluoroalkyl substances; BMI, body mass index; AHEI-P, alternative healthy eating index score in pregnancy.

© 2023 Zhang Y et al. *JAMA Network Open.*

eFigure 3. Spearman correlation coefficient matrix for early pregnancy dietary folate intake and plasma concentrations of per- and polyfluoroalkyl substances and folate among mother-singleton pairs in Project Viva.

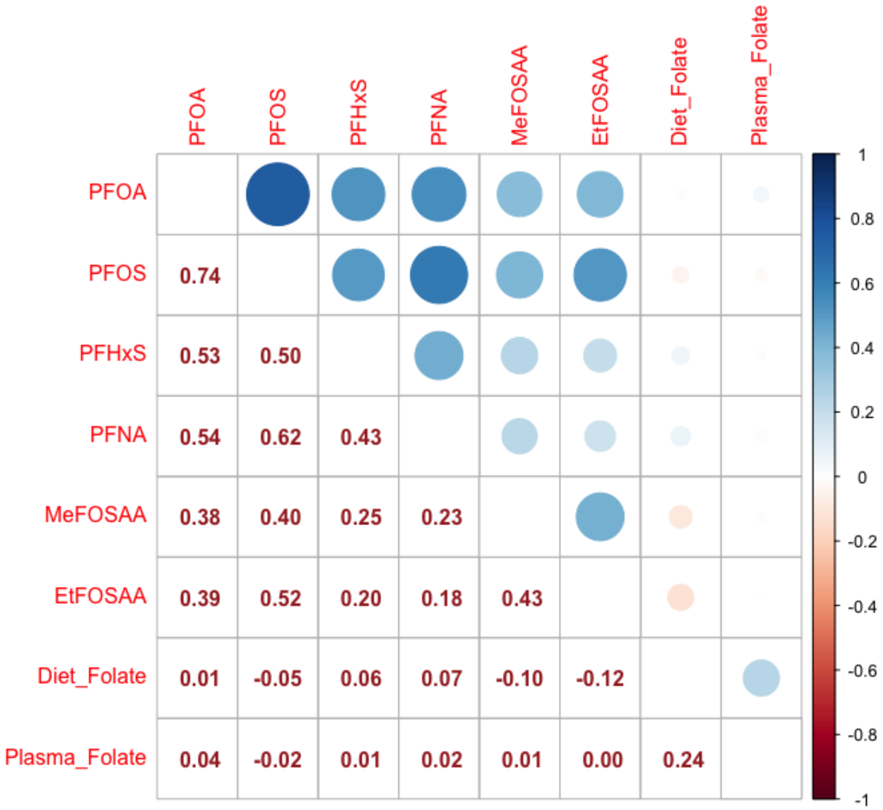

Abbrev. PFOA, perfluorooctanoic acid; PFOS, perfluorooctane sulfonic acid; PFHxS, perfluorohexane sulfonic acid; PFNA, perfluorononanoate; MeFOSAA, 2-(N-methyl-perfluorooctane sulfonamido) acetate; EtFOSAA, 2-(N-ethyl-perfluorooctane sulfonamido) acetate.

© 2023 Zhang Y et al. *JAMA Network Open.*

eFigure 4. Changes in the beta coefficients for the relationships between log-2 transformed plasma concentrations of per- and polyfluoroalkyl substances with birthweight across early pregnancy dietary folate equivalent intake.

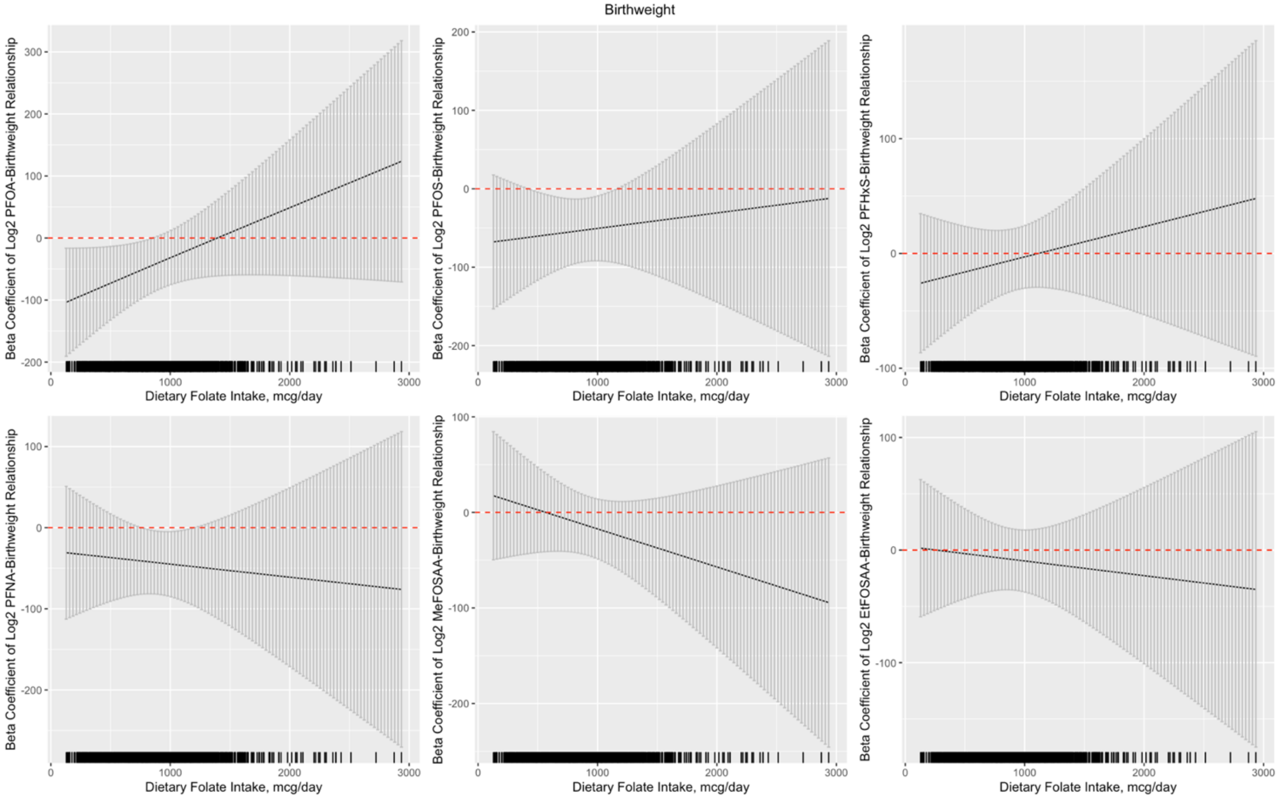

Abbrev. PFOA, perfluorooctanoic acid; PFOS, perfluorooctane sulfonic acid; PFHxS, perfluorohexane sulfonic acid; PFNA, perfluorononanoate; MeFOSAA, 2-(N-methyl-perfluorooctane sulfonamido) acetate; EtFOSAA, 2-(N-ethyl-perfluorooctane sulfonamido) acetate.
Note. N=2 outliers were excluded in the analyses with dietary folate equivalent intake > 3000 mcg/day.

© 2023 Zhang Y et al. *JAMA Network Open.*

eFigure 5. Changes in the beta coefficients for the relationships between log-2 transformed plasma concentrations of per- and polyfluoroalkyl substances with birthweight across early pregnancy plasma folate concentrations.

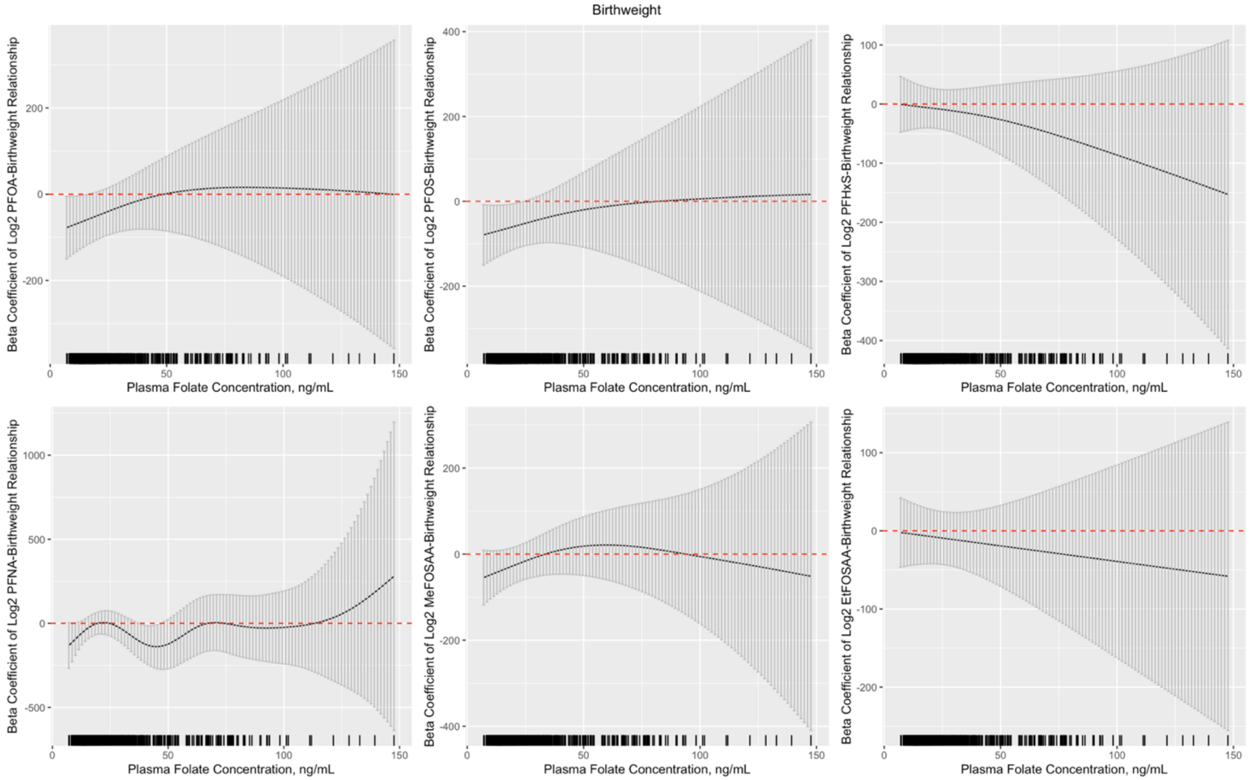

Abbrev. PFOA, perfluorooctanoic acid; PFOS, perfluorooctane sulfonic acid; PFHxS, perfluorohexane sulfonic acid; PFNA, perfluorononanoate; MeFOSAA, 2-(N-methyl-perfluorooctane sulfonamido) acetate; EtFOSAA, 2-(N-ethyl-perfluorooctane sulfonamido) acetate.
Note. N=3 outliers were excluded in the analyses with plasma folate concentrations > 150 ng/mL.

© 2023 Zhang Y et al. *JAMA Network Open.*

eFigure 6. Changes in the beta coefficients for the relationships between log-2 transformed plasma concentrations of per- and polyfluoroalkyl substances with birthweight z score across early pregnancy dietary folate equivalent intake.

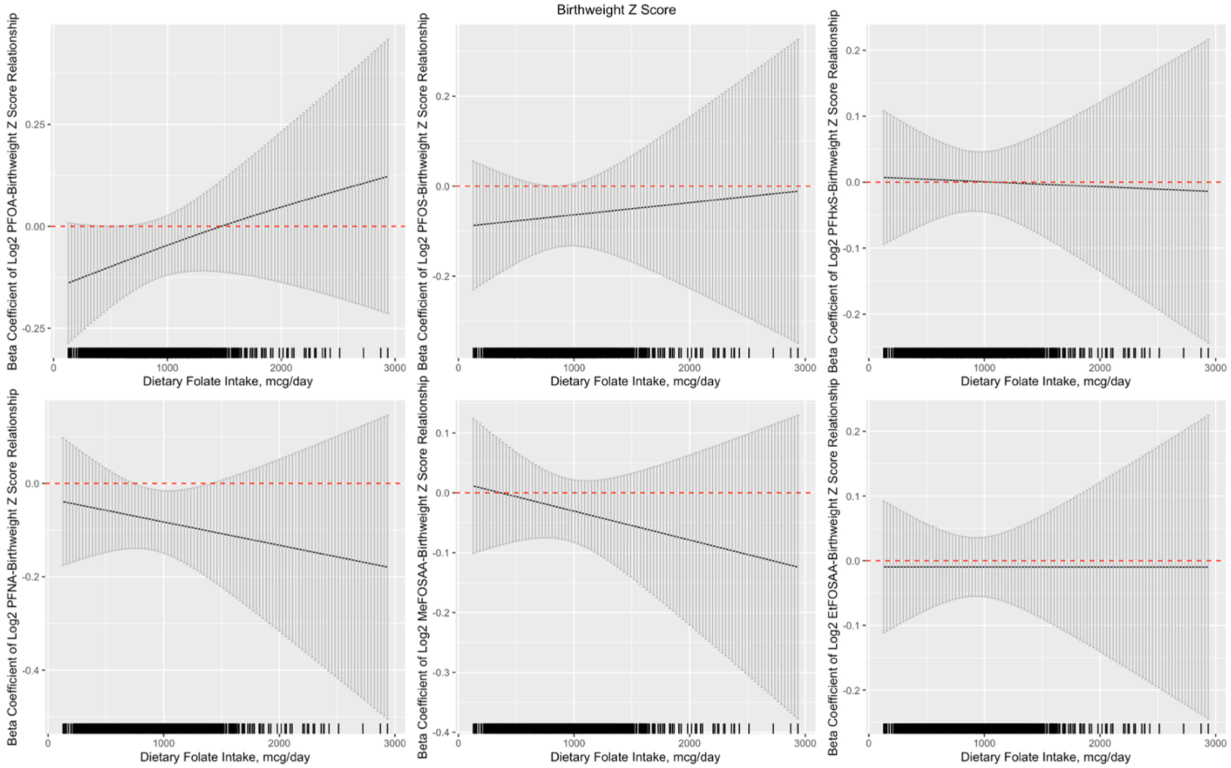

Abbrev. PFOA, perfluorooctanoic acid; PFOS, perfluorooctane sulfonic acid; PFHxS, perfluorohexane sulfonic acid; PFNA, perfluorononanoate; MeFOSAA, 2-(N-methyl-perfluorooctane sulfonamido) acetate; EtFOSAA, 2-(N-ethyl-perfluorooctane sulfonamido) acetate.
Note. N=2 outliers were excluded in the analyses with dietary folate equivalent intake > 3000 mcg/day.

© 2023 Zhang Y et al. *JAMA Network Open.*

eFigure 7. Changes in the beta coefficients for the relationships between log-2 transformed plasma concentrations of per- and polyfluoroalkyl substances with birthweight z score across early pregnancy plasma folate concentrations.

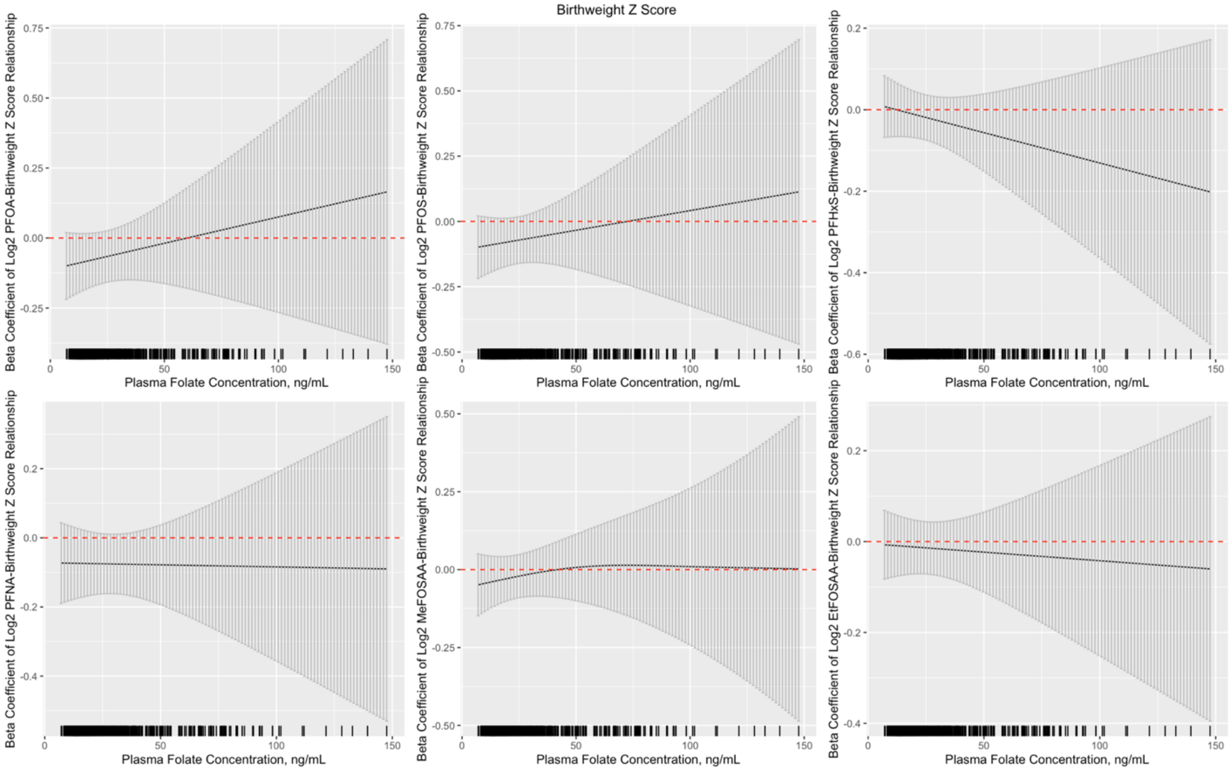

Abbrev. PFOA, perfluorooctanoic acid; PFOS, perfluorooctane sulfonic acid; PFHxS, perfluorohexane sulfonic acid; PFNA, perfluorononanoate; MeFOSAA, 2-(N-methyl-perfluorooctane sulfonamido) acetate; EtFOSAA, 2-(N-ethyl-perfluorooctane sulfonamido) acetate.
Note. N=3 outliers were excluded in the analyses with plasma folate concentrations > 150 ng/mL.

© 2023 Zhang Y et al. *JAMA Network Open.*

eFigure 8. Changes in log (odds ratio) for low birthweight in relation to log-2 transformed plasma concentrations of per- and polyfluoroalkyl substances across early pregnancy plasma folate concentrations.

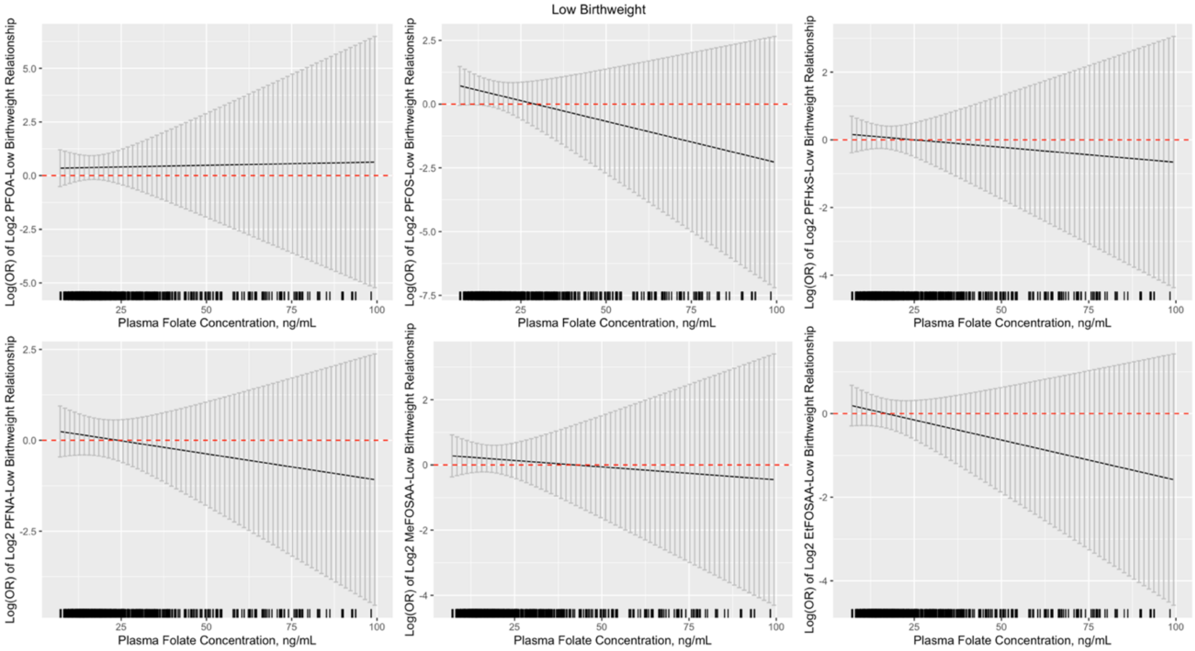

Abbrev. PFOA, perfluorooctanoic acid; PFOS, perfluorooctane sulfonic acid; PFHxS, perfluorohexane sulfonic acid; PFNA, perfluorononanoate; MeFOSAA, 2-(N-methyl-perfluorooctane sulfonamido) acetate; EtFOSAA, 2-(N-ethyl-perfluorooctane sulfonamido) acetate.

Note. N=3 outliers were excluded in the analyses with plasma folate concentrations > 150 ng/mL. Because of the large 95% CI confidence interval at high plasma folate concentrations due to sparse data, the figure only presented results at plasma folate concentrations < 100 ng/mL.

© 2023 Zhang Y et al. *JAMA Network Open.*

eFigure 9. Changes in the beta coefficients for the relationships between log-2 transformed plasma concentrations of per- and polyfluoroalkyl substances with gestational age across early pregnancy plasma folate concentrations.

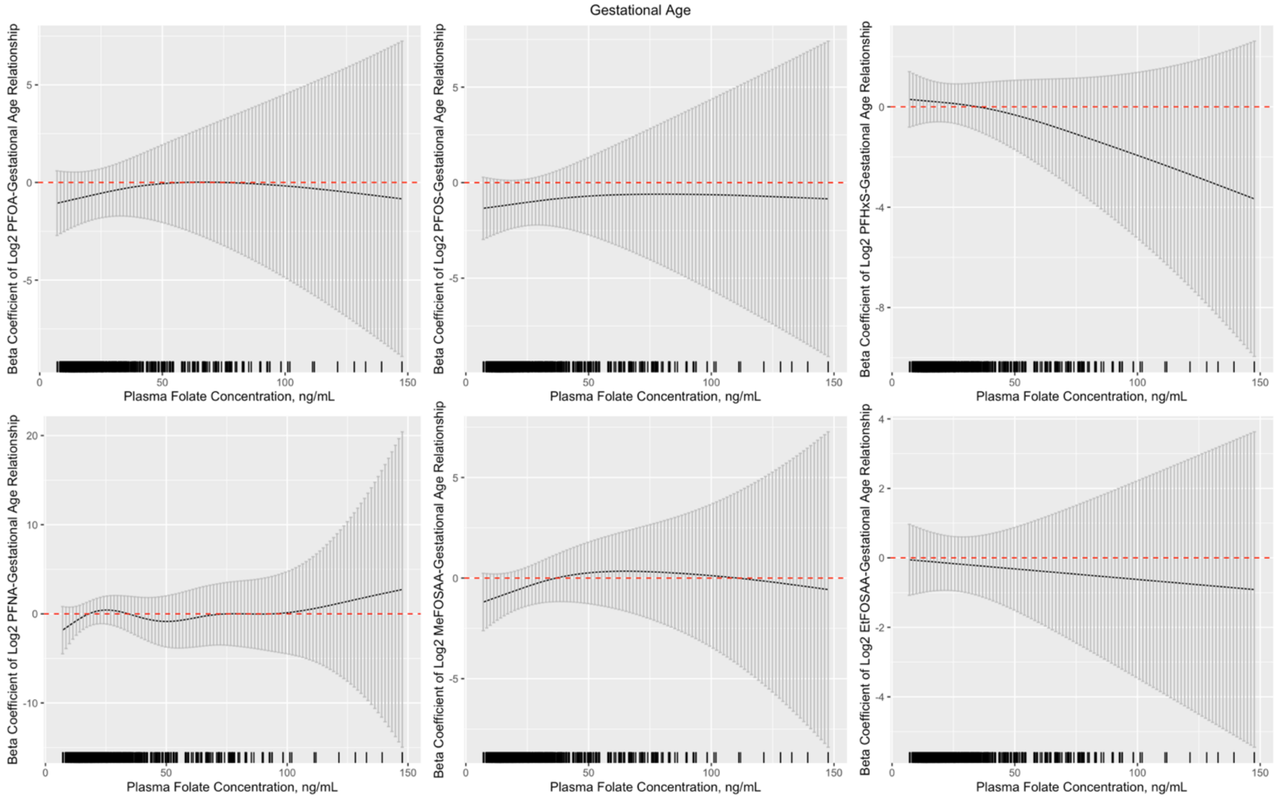

Abbrev. PFOA, perfluorooctanoic acid; PFOS, perfluorooctane sulfonic acid; PFHxS, perfluorohexane sulfonic acid; PFNA, perfluorononanoate; MeFOSAA, 2-(N-methyl-perfluorooctane sulfonamido) acetate; EtFOSAA, 2-(N-ethyl-perfluorooctane sulfonamido) acetate.
Note. N=3 outliers were excluded in the analyses with plasma folate concentrations > 150 ng/mL.

© 2023 Zhang Y et al. *JAMA Network Open.*

eMethods. Details on Analyses and Models

Analyses on preterm birth and low birthweight.

Secondary analyses were performed to assess the risks of preterm birth and low birthweight in relation to early pregnancy plasma PFAS concentrations across folate groups. Because of the low incidence of preterm birth (7.1%) and low birthweight (4.6%) in the study population and thus to preserve power in our analyses with the small number of cases, we dichotomized the sample into 1) below vs. above the lowest quartile of early pregnancy DFE intake; or 2) below vs. above the lowest quartile of plasma folate concentrations (in the subpopulation where plasma folate was quantified). These cut-points were informed *post-hoc* from associations with the continuous birth outcomes. We used logistic regression models as well as QGC to obtain the adjusted odds ratios (ORs) for preterm birth and low birthweight per doubling of plasma PFAS concentrations in the folate groups, adjusting for the above-mentioned covariates.

Generalized additive models.

As a complementary analysis, we additionally utilized generalized additive model (GAM) to explore the non-linear effect modification by folate (as DFE or plasma folate) on the relationship between individual PFAS compounds and each birth outcome. To simplify these analyses, we assumed a linear relationship between the log-2 transformed plasma PFAS concentration and birth outcomes. The GAM models allowed the beta coefficients of the linear PFAS-birth outcome associations to vary smoothly across the two continuous folate measures (DFE or plasma folate).

eResults. Details on Comparisons and Models

Comparisons of study population, subpopulation, with the original Project Viva cohort.

Compared with mothers of the total Project Viva cohort (N=2128), the study population (N=1400) and the subpopulation (N=898) included in the current analyses were slightly more likely to be White, have a college degree or higher, have annual household income > $70,000, plan for the index pregnancy, and report periconception folic acid supplement use, though the magnitudes of differences were small (eTable 1).

Results of generalized additive models.

The GAM models showed consistency in effect modification by folate status with findings from primary analyses in general. Specifically, the associations between PFAS and birth outcomes reported in the primary analyses [i.e., PFOA-lower birthweight (eFigures 4&5) and birthweight z score (eFigures 6&7), PFOS-lower birthweight (eFigure 5) and higher odds of low birthweight (eFigure 8)] were only present when DFE intake or plasma folate concentrations were low, but the associations became null as folate levels increased. The cut-offs of DFE or plasma folate concentration where the PFAS-birth outcome relationships changed to null were in general similar or higher than the lowest quartile levels. However, with GAM, we did not find effect modification by plasma folate on PFNA-birthweight (eFigure 5), and PFNA-gestational age (eFigure 9) relationships in contrast to primary analyses.

© 2023 Zhang Y et al. *JAMA Network Open.*
