# Supplementary material for: Association of Early Pregnancy Perfluoroalkyl and Polyfluoroalkyl Substance Exposure With Birth Outcomes
Source: JAMA Netw Open. 2023 May 31;6(5):e2314934. doi: 10.1001/jamanetworkopen.2023.14934 (PMC10233420; doi:10.1001/jamanetworkopen.2023.14934)
Supplement: Supplement 2. — Data Sharing Statement [file jamanetwopen-e2314934-s002.pdf]

Data Sharing Statement

Zhang. Association of Early Pregnancy Perfluoroalkyl and Polyfluoroalkyl Substance Exposure With Birth Outcomes. *JAMA Netw Open*. Published May 31, 2023. doi:10.1001/jamanetworkopen.2023.14934

Data
Data available: No

Additional Information
Explanation for why data not available: Data are available from the corresponding author upon reasonable request.
